# Paving the way for patient centricity in real-world evidence (RWE): Qualitative interviews to identify considerations for wider implementation of patient-reported outcomes in RWE generation

**DOI:** 10.1016/j.heliyon.2023.e20157

**Published:** 2023-09-14

**Authors:** Konrad Maruszczyk, Christel McMullan, Olalekan Lee Aiyegbusi, Thomas Keeley, Roger Wilson, Philip Collis, Catherine Bottomley, Melanie J. Calvert

**Affiliations:** a Centre for Patient Reported Outcome Research and Birmingham Health Partners Centre for Regulatory Science and Innovation, University of Birmingham, Birmingham, UK; b NIHR Blood and Transplant Research Unit (BTRU) in Precision Transplant and Cellular Therapeutics, University of Birmingham, Birmingham, UK; c NIHR Applied Research Collaboration (ARC) West Midlands, University of Birmingham, Birmingham, UK; d GlaxoSmithKline (GSK), Patient Centered Outcomes, Value Evidence and Outcomes, Brentford, UK; e Patient partner, UK; f Vitaccess Ltd., Oxford, UK; g NIHR Birmingham Biomedical Research Centre, University of Birmingham, Birmingham, UK

## Abstract

**Objectives:**

Real-world evidence (RWE) generation can be enhanced by including patient-reported outcomes (PROs). Methods for collecting and using PRO data in the real-world setting are currently underdeveloped and there is no international guidance specific to its use in this context. This study explored stakeholders' perspectives and needs for using PROs in RWE generation. Barriers, facilitators, and opportunities for wider use of PROs in real-world studies were also investigated.

**Methods:**

Online semi-structured interviews were conducted with international stakeholders: patients, patient advocates, regulators, payers, clinicians, academic researchers, and industry experts. Interviews were recorded, transcribed verbatim and analysed using NVivo 20. Thematic analysis was conducted based on the updated Consolidated Framework for Implementation Research (CFIR).

**Results:**

Twenty-three interviews were conducted. Participants confirmed that the use of PROs in RWE generation is not yet well established. Participants expressed a mixed level of confidence in the value of PROs collected in a real-world setting. Operational challenges associated with collecting routine PRO data to inform care delivery at the individual level (e.g., setting up infrastructure) need to be addressed. Methodological and other challenges (e.g., financing research) associated with collecting prospective *de novo* data in a real-world setting should be considered to facilitate PRO utilisation in real-world studies.

**Conclusions:**

Several opportunities and challenges were identified regarding the broader use of PROs in RWE research. Joint efforts from different stakeholders are needed to maximise PRO implementation, with consideration given to each stakeholders’ specific needs (e.g., by developing good practices).

## Highlights

• Methods for collecting and using PROs in the real-world setting are currently underdeveloped.
• This study shows that the current use of PROs in RWE generation is not yet well established. Participants expressed a mixed level of confidence in the value of PROs collected in a real-world setting. Barriers hampering the full implementation of PROs in RWE generation can be grouped as operational and methodological.
• Greater use of PROs in real-world settings can be achieved by addressing operational barriers to their use in routine care and by developing methodologies for the prospective collection of this type of data. Developing good practices for PRO data collection, analysis, and use in the real-world environment would help to maximise its benefits.

## 1 Introduction

Real-world evidence (RWE), as defined in Box 1, plays an increasingly prominent role in regulatory decisions, reimbursement and formulation of health policies [[1], [2], [3], [4], [5], [6]]. Analysis of real-world data (RWD) (see Box 1) can provide information about health interventions’ long-term tolerability, effectiveness and safety. Different study designs, including both prospective and retrospective data collection, could be used to generate RWE [7]. These studies are characterised by less stringent patient eligibility criteria than those required for registration purposes. Real-world studies allow for the investigation of diverse, large and heterogeneous patient populations. Recently published guidance and frameworks confirmed the growing interest in RWE globally. The US Food and Drug Administration (FDA) issued a framework for RWE [8], recently supplemented with four draft FDA RWE guidelines on data sources, standards, and regulatory considerations [[9], [10], [11], [12]]. In the UK, the National Institute for Health and Care Excellence (NICE) has published a RWE framework [13] and the Medicines & Healthcare products Regulatory Agency (MHRA) has issued two guideline documents focusing on using RWD to support regulatory decisions [14,15]. The Canadian HTA agency - Canadian Agency for Drugs and Technologies in Health (CADTH), has released a draft RWE guidance for public consultations [16]. These documents form foundations for greater use of RWE in regulatory and reimbursement decision-making. Moreover, there is an increase in sponsorship by the pharmaceutical industry of real-world, long-term safety studies [17].

Box 1 **Glossary**

Clinical outcome assessment (COA) [65] – a clinical evaluation instrument that is used to measure patient outcomes. There are four types of COAs: patient-reported outcomes, clinician-reported outcomes, observer-reported outcomes, and performance-based outcomes assessments.
Managed Access Programme [66] - a time-limited agreement that sets out conditions under which treatment will be reimbursed, including rules for data collection to address the uncertainties related to the effectiveness or cost-effectiveness of a treatment.
Patient-reported outcome (PRO) [21] - Reports of health status directly provided by patients, without interpretation by a clinician or anyone else. One of the types of COA.
Real-world data (RWD) [13] - Data relating to patient health or experience or care delivery collected outside the context of a highly controlled clinical trial. RWD can be routinely collected during the delivery of health or social care or can be collected prospectively to address specific research question(s). It can come from many different sources, including patient health records, administrative records, patient registries, surveys, observational cohort studies and digital health technologies.
Real-world evidence (RWE) [67] - Evidence generated from the analysis of real-world data.

Alt-text: Box 1

In the European Union (EU) the European Medicines Agency (EMA) and European medicines regulatory network have established the Data Analysis and Real World Interrogation Network (DARWIN EU) [18]. This data network will pull together medical information collected in routine practice from all EU countries. Its primary aim would be to inform the European regulatory decision-making process.

Other applications of RWD of interest to the wider scientific community include clinical trial tokenisation. Tokenisation enables to anonymously link multiple data sets providing comprehensive view of the patient journey while minimising risk of re-identification [19]. It is hoped to supplement clinical trials with data gathered in routine practice informing about trial participants’ health care service utilisation before, during and after the clinical trial formal follow-up period.

For years, regulators, patient advocates and health organisations have been postulating greater patient centricity in drug development and medical research. There have been numerous initiatives to put patients at the centre of the life science research and development processes. The US 21st Century Cures Act [20] addressed the need for more efficient delivery of treatments improving patient outcomes. One of the vehicles for greater patient centricity across the drug development lifecycle is patient-reported outcomes (PROs). PROs (see Box 1) – direct reports about patients’ health status without interpretation by a clinician or anyone else – are utilised at various stages of medical product development [21]. Until now, PROs have been mainly used in clinical trials [22,23]. PROs are also increasingly utilised in routine medical practice to inform healthcare decision-making at the individual level [24,25]. They have been shown to improve the quality of care and support shared clinical decision-making [26]. Moreover, numerous studies have demonstrated the positive impact of PRO use on patient satisfaction, health outcomes, patient-provider communication and disease management [[27], [28], [29], [30]]. Apart from being a useful tool in routine medical practice, PROs play an increasingly important role in regulatory and reimbursement decision-making, can facilitate healthcare quality improvement and inform decisions about financing healthcare services [[31], [32], [33]].

Despite this rapid development in the field, there is still a lack of widely accepted standards and best practices for utilising PROs in real-world studies [34]. Several guidelines on the implementation of PROs exist, but they mainly focus on randomised clinical trials (RCTs) or clinical practice [24,25,[35], [36], [37], [38], [39], [40], [41], [42], [43], [44]]. PRO guidance specific to the RWE context is fragmented and there is a lack of international guidelines [34]. The absence of universally accepted standards for using PROs in RWE generation is deemed a key factor for its underuse. A recent analysis of the clinicaltrials.gov database shows that PROs are underutilised in phase IV clinical studies compared to earlier stages of clinical research [45]. In addition to the lack of guidelines, triallists and other experts may experience other barriers to the use of PROs in RWE generation. A recent survey by the Professional Society for Health Economics and Outcomes Research (ISPOR) identified lack of transparency about the design of real-world studies and challenges with the analysis of PRO data collected in these studies, including approaches to dealing with missing data [46].

This qualitative study explores the stakeholders' perspectives on using PROs in a real-world setting. Specific objectives were to: (1) establish the current practice in the use of PROs in RWE generation, (2) identify stakeholders’ needs for use of PROs in real-world studies, (3) explore the perspectives of different stakeholders on the current and future practice of PRO use in RWE generation and (4) better understand barriers and facilitators for the use of PROs in real-world studies.

## 2 Materials and methods

The study and all study materials were approved by the Science, Technology, Engineering and Mathematics Ethical Review Committee at the University of Birmingham (Reference number: ERN_21–1240).

### 2.1 Methodology and methods

Qualitative methodology was selected for this study, as it allows better understanding of participants’ believes, behaviours, experiences and attitudes [[47], [48], [49]]. Semi-structured interviews were deemed an appropriate research method due to their ability to retain comparability between interviews. In addition, their relative flexibility allows for collecting in-depth data [47,[50], [51], [52]].

### 2.2 Participant selection

Participants were purposively recruited using two approaches. We approached leaders in PRO and RWE areas, who were identified during a previously conducted literature review [34]. Participants were also recruited utilising the existing University of Birmingham Centre for Patient Reported Outcome Research's (CPROR) networks and contacts. Invitation emails, participant information, and consent forms were sent out to the potential participants. Interviews were subsequently scheduled with those who accepted our invitations and provided written informed consent. Two types of participants have been distinguished: patient experts [53,54] (patients and patient advocates - individuals associated with patient groups or organisations that represent and support patients and their families living with a specific condition) and other experts (academic researchers, regulators, payers and industry experts). Patient experts were offered £20 vouchers as reimbursement for their participation.

### 2.3 Data collection

Semi-structured in-depth interviews were conducted using topic guides prepared by the study team. Two separate topic guides were developed – one for patient experts (Appendix 1) and one for other experts (Appendix 2). Topic guides were formulated based on the findings from previously published systematic reviews and discussions within the research team [34,45].

Interviews were conducted between February and October 2022 and held online using Zoom™. Conversations lasted between 40 and 90 min. Two pilot interviews were conducted by KM and observed by a second researcher (CM) to check the effectiveness of the topic guide in eliciting the required information. All conversations were audio-recorded and transcribed verbatim using Zoom's built-in features. Transcripts were then checked for correctness and amended where necessary by KM.

### 2.4 Data analysis

#### 2.4.1 Analytical framework

**Fig. 1:**
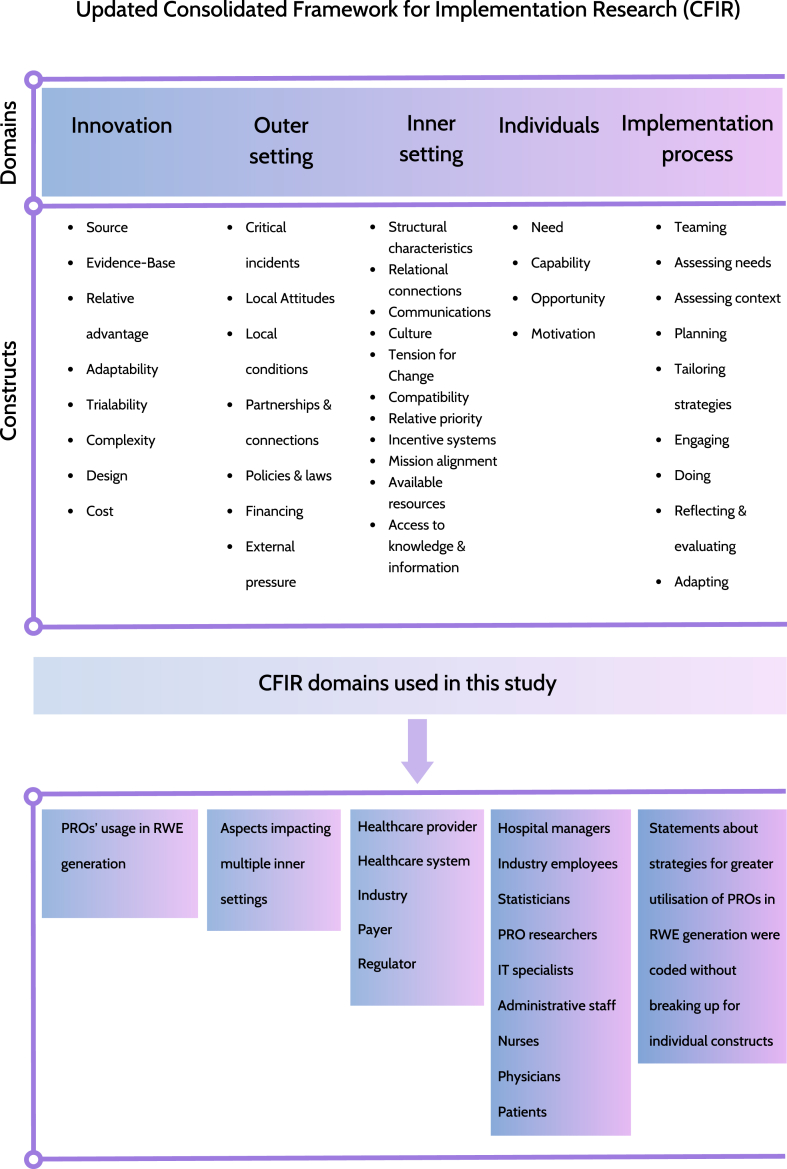
CFIR conceptual framework (adapted from Damschroder et al.) Legend: CFIR, Consolidated Framework for Implementation Research; IT, information technology; PRO, patient-reported outcomes; RWE, real-world evidence.

The updated Consolidated Framework for Implementation Research (CFIR) underpinned data collection and analysis (Fig. 1) [55]. The CFIR framework is a commonly used data extraction tool for the characterisation of the determinants of effective implementation of innovations in healthcare [56]. It provides a comprehensive framework of constructs, which can be consistently used for systematic analysis and organisation of diverse data.

The CFIR framework consists of five domains: (I) Innovation, (II) Outer setting, (III) Inner setting, (IV) Individuals and (V) Implementation process. We used the CFIR to increase the richness of our data analysis. However, not all of the constructs were applicable to our data and so were not used for analysis and presentation of findings.

#### 2.4.2 Coding process and analysis

Two coders were involved in this work: KM coded all transcripts, and CM coded a randomly selected sample of four interview transcripts (17%). CM is a highly experienced qualitative researcher, who leads and teaches qualitative research methods courses. KM completed a qualitative research methods module for postgraduate students at the University of Birmingham before approaching this work. Both coders initially worked independently. Subsequently, they compared and discussed codes assigned to statements of individual participants. Discrepancies were discussed by the two researchers and consensus was achieved. Interview transcripts were coded using QSR NVivo 20 software [57]. A coding framework drawing on the updated CFIR [55] was used to analyse transcripts deductively. Further new constructs (five within “Inner settings” and nine within “Individuals” domains) were added in through data engagement and the analytical process.

### 2.5 Investigator triangulation

Preliminary findings of the qualitative analysis were presented to the patient partner (RW) and an industry professional with hands-on experience in setting up real-world studies (CB). They reflected on the data, discussed and provided their interpretations of the key themes identified [[58], [59], [60]]. Their contributions were incorporated into the results and discussion presented in this manuscript. Investigator triangulation was conducted to decrease the researcher's influence on the interpretation of gathered data, diversify the interpretation by using different perspectives and enhance the credibility of findings [58].

## 3 Results

### 3.1 Participants

Twenty-three semi-structured in-depth interviews were conducted as part of this study. Seven patient experts and sixteen academic researchers, regulators, payers, and industry experts consented. Characteristics of interview participants are summarised in Table 1.

**Table 1:** Characteristics of participants.

	Patient experts (N = 7)	Other experts (N = 16)
Country		
European Union	–	3
Canada	–	3
United Kingdom	7	4
United States	–	6
Role		
Regulator	–	5
Academic researcher	–	7
Payer	–	1
Industry expert	–	3
Patient	5	–
Patient advocate	2	–
Gender		
Male	5	6
Female	2	10

### 3.2 Key themes

Our analysis identified themes which are presented according to the updated CFIR [55] domains. These themes and illustrative quotations are presented in Appendix 3.

Not all the updated CFIR constructs were relevant to our study, and only those applicable are included in the comprehensive results table (Appendix 3). This study defined innovation of interest as “PROs’ usage in RWE generation”. Five “Inner settings” were identified and described, including: “Healthcare provider”, “Healthcare system”, “Industry”, “Payer” and “Regulator”. Additionally, roles subdomains under the “Individuals” domain was altered with stakeholders applicable to our topic (hospital managers, industry employees, statisticians, PRO researchers, information technology (IT) specialists, administrative staff, nurses, physicians, and patients). Moreover, we did not distinguish individual constructs under the “Implementation process” domain as its collateral constructs were deemed not applicable to the collected data.

**Fig. 2:**
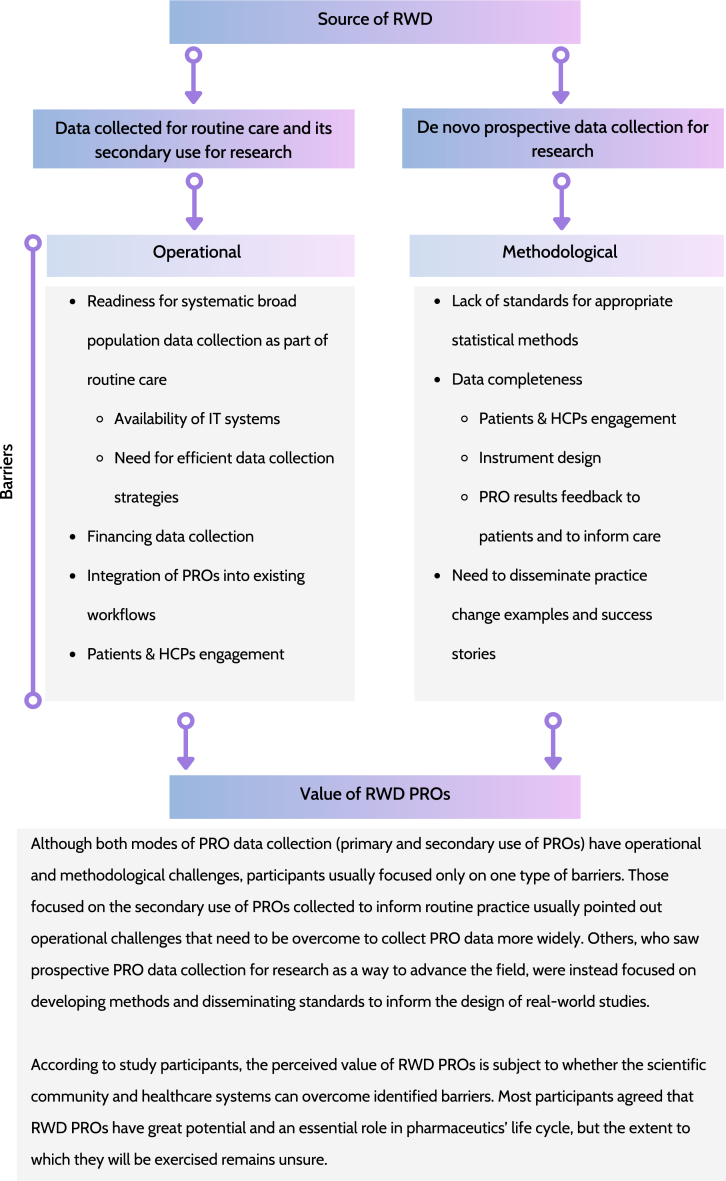
Key study findings: modes of PRO data collection, barriers for its full implementation and impact on their perceived value Legend: HCPs, healthcare professionals; IT, information technology; PROs, patient-reported outcomes; RWD, real-world data.

The remaining of the results section describes key themes, formed by data captured in multiple domains of the updated CFIR (Fig. 2). Those themes compose the most important study findings and addresses following issues:

• sources of RWD,
• value of PROs,
• data collection as part of routine care,
• prospective data collection,
• increase in the use of PROs in routine care,
• facilitating prospective real-world studies,
• patient engagement,
• instrument design, and
• good practices dissemination.

### 3.3 Sources of RWD^1^

There was no agreement about the universal definition of RWD between participants (quote #37). Less than half particiapnts, mainly those with a background in academia, used a narrow definition of RWD. They considered RWD as data captured routinely as part of claims databases or electronic health records (EHRs) only. In their opinion, prospective data collection conducted as part of the study with an *à priori* research question does not fulfil the definition of RWD (quotes #39 and #40). In contrast, others (mainly industry or consultancy employees, payers and regulators) used a more inclusive RWD definition. They referred to the spectrum of real-world data sources: registries, patient surveys, observational studies and pragmatic trials.

Participants with a more conservative view on sources of RWD pointed out that participation in a study with prospective data collection has to be preceded by obtaining patient consent (quote #41). They saw selection bias imposed by the requirement of obtaining patients’ informed consent as a threat to the generalisability of study findings (quote #38). Moreover, they argued that the characteristics of individuals who agree to participate in studies tend to differ from those of the general population (quote #57). Thus, data collected prospectively should not be characterised as a “real-world”.

Differences in the perception of RWD sources have implications for recommendations given by participants on advancing the field. Participants who limited their definition of RWD to data collected primarily in routine care and then re-used for research, mainly focused on barriers hampering routine PRO data collection. They believed that a lack of routine PRO data collection is the main issue which needs to be addressed in the first place, and this was seen a necessary condition to advance the field (#quote 223). On the other hand, participants who adopted a broader RWE definition (quote #42) allow, at minimum, for prospective data collection, e.g., supplementing data captured in EHRs. Their recommendations were more wide-reaching and not limited to operational barriers to data collection in routine care. They commented on challenges associated with running prospective real-world studies and limited acceptance of evidence generated in this way due to a lack of widely accepted standards and requirements (quote #108).

### 3.4 Value of PROs^2^

Almost all participants acknowledged multiple potential applications for PROs in RWE generation (quote #7). Efficiencies associated with collecting PRO data were realised through their use to inform individual patient care and building up an evidence base to support decision-making processes, e.g. treatment option selection (quotes #11 and #12). It was observed that different participant types had different expectations with respect to PROs’ value. Most of the patient experts saw PROs as a tool that, in the first place, can inform their care – utilising their responses for disease progression monitoring or as a vehicle for building up evidence to help other patients with similar conditions in the future to choose the most appropriate treatment method (quotes #200 and #206). Moreover, they were seen as a valuable tool for self-diagnosis and regaining power over their healthcare (#quote 18). Clinicians often saw PROs as a means to identify patients who require special attention and improve care of individual patients (quotes #19 and #181–183). All regulators and payers mentioned the potential of PROs collected in the real-world in supplementing evidence currently used to inform their decisions (quotes #73 and #152).

Nevertheless, statements from five participants pointed out that the PRO RWD field is not yet well established. Interviewees realised that it is difficult to assess how big a role PROs will play in the RWE space as we are still very early with its implementation for this purpose (quote #72). Moreover, participants with a background in regulatory and payer organisations were unsure how exactly PROs collected in the real-world setting could informed their decisions due to the lack of guidance (quote #148).

### 3.5 Data collection as part of routine care^3^

The primary problem perceived by the participants in this area is the lack of routine data collection in most jurisdictions (quote #97). Less than half participants highlighted that efforts need to be made to initiate data collection in selected sub-populations of interest. They noticed that appropriate infrastructure must be in place to support data collection in routine practice (quote #98). It usually requires large-scale implementation, often at the health system level (quote #78). Thus, more than half of the participants identified operational and infrastructural barriers hampering the introduction of PROs associated with a lack of appropriate IT systems, funding or integrating data collection into current workflows and practices (quotes #75, #127, #134 and #136). Some participants emphasised that the primary reason for collecting PROs is to inform routine medical practice at the individual level. PRO data are then recorded as part of EHRs and can be later re-used for research purposes (quote #40). A few participants, however, noted that PRO data are rarely collected routinely (quote #97). Additionally, concerns about the suitability of PRO instruments used to inform individual decision-making for answering specific research questions of interest were raised (quote #217) by a few participants.

### 3.6 Prospective data collection^4^

The majority of participants admitted that despite increasing interest in their use, PRO data are still rarely collected to inform routine care (quote #133). Even if they are, it is not clear whether PRO instruments selected primarily to inform care at the individual level would be able to answer the research question pertaining to RWE generation. Industry and consultancy employees described that usually, EHRs do not hold all necessary information (e.g., PRO data not collected or unsuitable PRO instrument used, timing of assessments does not allow to address research question, missing data), and most of the time, some form of prospective data collection is required. A few participants acknowledged that in the case of prospective data collection, there is no need for a lengthy and challenging process of delivering infrastructural change at the health system level to ensure that appropriate tools for PRO data collection are in place. Instead, the study sponsor is responsible for recruiting sites eager to participate in the research project and finances the site(s) for data collection. In that case, sponsors also coordinate the implementation of necessary IT tools (quotes #80 and #118). Participants noticed that PRO responses obtained in this way might (quote #11) or might not (quote #128) be fed back to the clinicians and be actively used in the care delivery for study participants.

### 3.7 Increase in the use of PROs in routine care^5^

Several barriers that must be addressed to allow for the more common use of PROs in routine medical practice were reported. First, large-scale infrastructure changes are required (quotes#134 and #137). Less than half participants noted that policymakers could mandate or incentivise PRO data collection (quotes #111 and #112). The public sector was also often mentioned to be involved in financing data collection to some extent (quotes #111, #115 and #116). Additionally, it was often mentioned that the successful implementation of PROs into clinical workflows requires a change of behaviour from healthcare professionals (HCPs) (quote #130). Their buy-in and actual utilisation of PROs, e.g. by discussing patient responses, impacts patients’ willingness to provide data (quote #176). Several approaches can be taken to encourage patients to provide data, including educating them about the value of PROs and informing them about the purpose of data collection (quotes #201, #205 and #219).

A few participants postulated that the successful implementation of PROs into clinical practice should be facilitated by creating an ecosystem of highly engaged HCPs – champions, who help demonstrate the value of PROs to their peers (quote #184). More than a half participants believed that HCPs must acknowledge the importance of using PROs in routine care (quote #180) to allow for its successful use in clinical practice. Their positive attitude should keep patients motivated and help sustain their long-term reporting (quote #196). Moreover, around a quater of participants mentioned that realising the benefits of PROs in individual's care should, on the other hand, motivate patients to report (quote #206). Data gathered in this way can be then re-used in RWE generation (quote #222). According to less than half participants, this evidence can be used to better inform the care of future patients struggling with similar conditions (quote #12). Almost of participants realised that making it happen is not easy and straightforward (quote #71). They noticed that successful implementation strongly depends on the involvement of health institutions, which are often rigid, and getting them on board might be tedious (quote #220). On the other hand, a few participants argued that guidelines and standards for PRO use in routine care are already available [24,25,44,61,62], which should enhance the use of PROs in routine care and indirectly accelerate PROs implementation for RWE generation as well.

### 3.8 Facilitating prospective real-world studies^6^

A commonly mentioned barrier hampering the broader use of prospective real-world studies is the lack of guidelines and well-established standards for their implementation (quotes #89–90 and #108). Success stories demonstrating the added value of PROs collected in real-world settings are needed (quote #227), according to majority of study participants. They argued that practice change resulting from these type of studies could help convince a wider scientific audience about their usefulness (quotes #92 and #226). Less than half participants added that lessons learned along successful PRO implementations should be reflected in emerging good practices e.g. decision-makers’ guidelines informing how RWE can influence their decisions (quotes #109–110 and #225). Majority of industry and consultancy employees stated that running prospective studies seems a more manageable task, which does not entail changes at the health system level (quote #80). A few participants reported that the industry is interested in sponsoring this type of evidence generation, and there is a growing number of successful completed studies of this design (quote #118). At the same time companies struggle to use this evidence in regulatory or reimbursement processes (quote #110). They noticed that the value of this type of evidence was not fully understood yet, and thus decision-makers are reluctant to accept it (quotes #88–92).

### 3.9 Patient engagement^7^

Issues around patient engagement and burden apply to both primary and secondary use of PRO data and were frequently mentioned by participants. Majority of participants emphasised that patients should be engaged at every step of the study (quote #201). They are vital in setting up the research, selecting PRO instruments and proselytising participation (quote #204). A few participants stated that PRO instruments used for data collection must be relevant to the participants’ health states to facilitate long-term patient retention (quotes #189–191). Participants shared several recommendations that should be used when designing and implementing studies. Firstly, the time needed to complete questionnaires and the frequency of data provision should be considered as it impacts patients' burden and willingness to provide data (quotes #188 and #195). Secondly, in general patients are eager to share their experiences and provide the study with their data (quotes #197–199). Besides informing their care, PRO data provision is often motivated altruistically (quotes #206 and #200). Patient experts often mentioned that the awareness of contributing to the ongoing research and helping patients with similar health problems is an important motivator for providing their data. Participants pointed out that patients should be informed about the purpose of data collection and study progress (quote #201). Patient experts frequently mentioned that receiving feedback with study results is a significant incentive to continue providing data (quote #205). They also mentioned that updating them about the progress of the study, is often perceived as a form of thanking participants for their involvement in the research. Moreover, majority of participants stressed that it is crucial to use collected data and act upon them if made available to the health teams (quote #50).

### 3.10 Instrument design, selection and administration^8^

Patient experts shared considerations for designing and selecting PRO instruments in the real-world setting. Firstly, tools for data collection should be compatible with different operating systems and device types (laptop, tablet, mobile) (quote #211). Secondly, patient-reported outcome measures (PROMs) should have an attractive and easy-to-follow layout (quote #212). Patient experts also often pointed out that questions should be simple, free from spelling mistakes and without extensive use of abbreviations. They also often mentioned that electronic data capture should be supported by automated reminders, as it helps keep participants engaged and reduces the number of missing measurements (quote #215).

### 3.11 Good practices dissemination^9^

Less than half of the participants highlighted that educating stakeholders across the board is essential for successful implementation of PROs in RWE generation. Patients, regulators, payers and industry workers should be informed about the value of PROs in the real-world setting and the benefits associated with different use cases of PROs (quote #219). Participants frequently mentioned that international scientific societies can be an excellent platform to share experiences and emerging good practices for running real-world studies. A few participants argued that examples of methodologically robust studies leading to practice change should convince regulators and payer that their decisions can be made in greater extend based on RWE (quotes #226–227). As reported by a few participants this should ultimately lead to development of guidance and setting up acceptable level of evidence for different use cases for regulatory and reimbursement decision-making.

## 4 Discussion

This study revealed the attitudes of stakeholders towards using PROs in the RWE generation. Participants listed multiple potential applications for PROs in a real-world setting. Nevertheless, numerous barriers hampering the broader use of PROs were also identified. Mixed opinions about the value of PROs in RWE generation were present, indicating that the field is still in the early stages of development.

Barriers can be grouped as operational and methodological challenges, and they must be addressed to advance the field to exercise the full benefits of PROs in RWE generation. Operational challenges associated with PRO data collection in routine care include: setting up systems for collecting PRO data from broad populations as part of their routine care, implementing efficient data collection strategies, ensuring appropriate financing is in place, building up IT infrastructure, and integrating data collection into existing workflows. Methodological issues mentioned were often focused on strategies for dealing with missing data. Efforts should be made to maximise the completeness of the gathered data sets (participant and HCPs engagement, result feedback to patients, etc.). Statistical methods for dealing with missing data exist and can be successfully carried over from the clinical trial environment, but guidance is needed to indicate which methods are acceptable in particular use cases. The need for guidance for more appropriate use of Clinical Outcome Assessments (COAs), including PROs, to meet challenges present in real-world studies was also recently mentioned by Rylands and colleagues [46]. They acknowledged that robust study design to guide selection, analysis, interpretation and integration of COAs is of critical importance for generating high-quality, fit-for-purpose and meaningful RWE, which is in line with our findings. Thus, standards should be set to avoid confusion about the analytical approaches used. Widely accepted methodological standards and data collection practices should be reflected in the emerging good practices.

The lack of consensus about the definition of RWE was also reported in a study where regulators and payers were interviewed [63]. The lack of agreement on the study setting constituting RWE hampers guidance development and advancing the field. Ambiguity around RWE term can also lead to misunderstandings between different healthcare stakeholders [63]. Calvert et al. [64] summarised priorities which need to be addressed to allow for greater inclusion of PROs in RWE. Challenges reported by participants of this study overlap to great extent with those priorities.

Our findings identified two areas of focus to facilitate utilisation of PROs in real-world studies. The first is to address operational challenges associated with collecting routine PRO data to inform care delivery at the individual level. The second is to focus on addressing methodological and other challenges related to studies collecting prospective *de novo* data in real-world setting.

Another frequently mentioned issue which needs to be addressed is how to fund PRO data collection. Multiple possible funding entities were mentioned by participants – government, payers, pharma companies and healthcare providers. Several models of financing PRO data collection are possible and should be explored in future research. The collection of PROs as part of managed access programs (as described in glossary, Box 1) poses a promising opportunity for its broader utilisation. PROs might provide a valuable source of information for re-evaluating a drug when the initial reimbursement decision was burdened with uncertainties around meaningful endpoints to patients. Additionally, post-authorisation safety and tolerability studies were highlighted as those with a potential for substantial PRO usage in a real-world setting.

Further research is needed to determine the value of PROs collected in real-world settings for various use cases. Practice-changing studies are required to demonstrate the full potential of PROs. Efforts of regulators, payers and the broader scientific community are needed to guide how this type of data should be collected, analysed, interpreted and integrated to provide robust answers to questions asked by different stakeholders. Most likely future standards for using this type of data will depend on the study setting and sources of RWD. Thus, recommendations may need to be tailored to specific use cases of PROs collected in a real-world environment.

## 5 Strengths

Our study recruited participants representing various roles, organisation types and viewpoints. Due to that, we were able to gather a rich data set suitable for meaningful qualitative analysis. Different perspectives and opinions were presented, allowing for identifying areas lacking agreement where additional research is needed.

## 6 Limitations

Given the size of the study sample, the findings may not represent the views of all stakeholder group. However, the qualitative nature provides the opportunity to explore in depth the views of participants in a manner that would not be possible using quantitative methods such as surveys. Most of the individuals recruited for the study have a keen interest in PROs or are professionally engaged in RWE. This makes them valuable and knowledgeable sources of information about issues in the scope of this research. But it can introduce bias as our interviewees might present a more favourable outlook on PROs in the RWE generation than the broader clinical research community. Furthermore, patient experts participating in this study were recruited solely in the UK, which might limit the generalisation of our findings in other geographic locations.

## 7 Conclusions

The use of PROs in RWE generation is not well established yet. Several opportunities and challenges were identified regarding the broader use of PROs in RWE research. A mixed level of confidence about the value of PROs collected in a real-world setting is present among participants. Barriers hampering the full implementation of PROs in RWE generation can be grouped as operational and methodological. The needs of various stakeholder groups (including patients, HCPs, regulators, payers, and industry) should be considered when implementing PROs. Setting good practices for PRO data collection, analysis, and use in the real-world would help to maximise its benefits.

## Ethics statement

The study and all study materials were approved by the Science, Technology, Engineering and Mathematics Ethical Review Committee at the University of Birmingham (Reference number: ERN_21–1240). Informed consent was obtained from all study participants.

## Funding

This research was conducted as part of a PhD programme funded by GSK Ltd. Dr Keeley is a co-supervisor of Mr Maruszczyk (the holder of the GSK PhD grant) and is a Director at GSK Ltd. In his role as co-supervisor Dr Keeley inputted to all stages of this research.

## Contributorship

Konrad Maruszczyk, Christel McMullan: Conceived and designed the experiments; Performed the experiments; Analyzed and interpreted the data; Contributed reagents, materials, analysis tools or data; Wrote the paper.

Olalekan Lee Aiyegbusi, Thomas Keeley, Melanie J Calvert: Conceived and designed the experiments, Contributed reagents, materials, analysis tools or data; Wrote the paper.

Roger Wilson, Philip Collis, Catherine Bottomley: Analyzed and interpreted the data; Wrote the paper.

## Declaration of competing interest

The authors declare the following financial interests/personal relationships which may be considered as potential competing interests:Konrad Maruszczyk reports financial support was provided by GSK plc. Melanie Calvert reports financial support was provided by GSK plc. Christel McMullan reports financial support was provided by GSK plc. Olalekan Lee Aiyegbusi reports financial support was provided by GSK plc. Melanie Calvert reports a relationship with NIHR Birmingham Biomedical Research Centre that includes: funding grants. Melanie Calvert reports a relationship with Health Data Research UK that includes: funding grants. Melanie Calvert reports a relationship with Innovate UK that includes: funding grants. Melanie Calvert reports a relationship with Macmillan Cancer Support that includes: funding grants. Melanie Calvert reports a relationship with GSK plc that includes: consulting or advisory and funding grants. Melanie Calvert reports a relationship with UCB Pharma SA that includes: funding grants. Melanie Calvert reports a relationship with Research England that includes: funding grants. Melanie Calvert reports a relationship with European Commission and EFPIA that includes: funding grants. Melanie Calvert reports a relationship with Brain Tumor Charity that includes: funding grants. Melanie Calvert reports a relationship with Gilead Sciences Inc that includes: funding grants. Melanie Calvert reports a relationship with Janssen Pharmaceuticals Inc that includes: funding grants. Melanie Calvert reports a relationship with National Institute for Health and Care Research that includes: funding grants. Melanie Calvert reports a relationship with UK Research and Innovation that includes: funding grants. Melanie Calvert reports a relationship with Aparito that includes: consulting or advisory. Melanie Calvert reports a relationship with CIS Oncology that includes: consulting or advisory. Melanie Calvert reports a relationship with Takeda UK Ltd that includes: consulting or advisory. Melanie Calvert reports a relationship with Merck & Co Inc that includes: consulting or advisory. Melanie Calvert reports a relationship with Daiichi Sankyo Inc that includes: consulting or advisory. Melanie Calvert reports a relationship with Glaukos Corporation that includes: consulting or advisory. Melanie Calvert reports a relationship with Patient-Centered Outcomes Research Institute that includes: consulting or advisory. Melanie Calvert reports a relationship with Genentech that includes: consulting or advisory. Melanie Calvert reports a relationship with Vertex that includes: consulting or advisory. Melanie Calvert reports a relationship with Icon plc that includes: consulting or advisory. Melanie Calvert reports a relationship with University of Maastricht that includes: speaking and lecture fees. Melanie Calvert reports a relationship with Cochrane Portugal that includes: speaking and lecture fees. Melanie Calvert reports a relationship with South-Eastern Norway Regional Health Authority that includes: paid expert testimony. Melanie Calvert reports a relationship with Singapore National Medical Research Council that includes: paid expert testimony. Melanie Calvert reports a relationship with PROTEUS Consortium that includes: board membership. Catherine Bottomley reports a relationship with Vitaccess Limited that includes: employment. Olalekan Lee Aiyegbusi reports a relationship with GSK plc that includes: consulting or advisory and funding grants. Olalekan Lee Aiyegbusi1 reports a relationship with Merck & Co Inc that includes: consulting or advisory. Christel McMullan reports a relationship with National Institute for Health and Care Research that includes: funding grants. Christel McMullan reports a relationship with CIS Oncology that includes: funding grants. Christel McMullan reports a relationship with Aparito that includes: consulting or advisory. Thomas Keeley reports a relationship with GSK plc that includes: employment and equity or stocks.

## Footnotes

1 This subsection contains data grouped under the following CFIR constructs: Innovation domain (Innovation complexity), Outer setting domain (Policies & Laws), Implementation process domain.

2 This subsection contains data grouped under the following CFIR constructs: Innovation domain (Innovation Relative Advantage, Innovation complexity), Individuals domain (Patients(Motivation), Physicians(Motivation)), Inner settings domain(Regulator(Work infrastructure), Payer(Compatibility)).

3 This subsection contains data grouped under the following CFIR constructs: Innovation domain (Innovation cost, Innovation complexity), Outer setting domain (Local conditions), Inner settings domain (Healthcare provider (IT infrastructure, Funding).

4 This subsection contains data grouped under the following CFIR constructs: Innovation domain (Innovative relative advantage), Outer setting domain (Financing), Inner settings domain (Healthcare provider(Compatibility, IT infrastructure)).

5 This subsection contains data grouped under the following CFIR constructs: Innovation domain (Innovation Relative Advantage, Innovation Complexity), Outer setting domain (Policies & Laws, Financing), Inner settings domain (Healthcare provider(Funding, IT infrastructure, Work infrastructure)), Individuals domain (Physicians(Opportunity, Motivation), Patients(Motivation)), Implementation process domain.

6 This subsection contains data grouped under the following CFIR constructs: Innovation domain (Innovation cost), Outer setting domain (Local attitudes, Policies & laws, Financing), Implementation process domain.

7 This subsection contains data grouped under the following CFIR constructs: Innovation domain (Innovation Complexity), Individuals domain (Patients (Motivation, Capability, Opportunity)).

8 This subsection contains data grouped under the following CFIR constructs: Implementation process domain.

9 This subsection contains data grouped under the following CFIR constructs: Implementation process domain.
