# Supplementary material for: Paving the way for patient centricity in real-world evidence (RWE): Qualitative interviews to identify considerations for wider implementation of patient-reported outcomes in RWE generation
Source: Heliyon. 2023 Sep 14;9(9):e20157. doi: 10.1016/j.heliyon.2023.e20157 (PMC10559915; doi:10.1016/j.heliyon.2023.e20157)
Supplement: Multimedia component 1 [file mmc1.docx]

Appendix 1. Patient experts interview topic guide.

**Introduction:**

Introduce self as a UoB PhD student and that this interview is being undertaken as part of the research project funded by unrestricted educational research grant from GSK.

**Study recap (general purpose of the interview):**

Patient-reported outcomes (PROs) represent health status as reported directly by the patient, without interpretation by a clinician or anyone else. PROs are often collected in trials to understand the impact of disease and treatment on patient symptoms and quality of life. They can be used to help assess if a treatment is safe and tolerable. Once drugs have been tested in trials and approved by regulators for use we still want to know about how effective the therapy is, and if it is safe as it is used in the broader target population. This is called real-world evidence (RWE).

PROs are usually collected via questionnaires that elicit information about symptoms, physical functioning and/or health-related quality of life. The objective of this study is to better understand the use of this type of health questionnaires in the long-term studies of drugs following the completion of clinical trials. Today, we would like to find out more about your views about collecting this information in routine medical practice to assess if a treatment that have been approved for use are working as expected.

Health questionnaires can be completed directly or remotely, using paper, mobile apps, telephone or being asked by health care staff and recorded in patients’ health records.

**Consent**

Check that the respondents are still happy to take part and have signed the consent form. Participants will be reminded that all individual self-identifiers will be removed before transcripts are analysed and that they can stop the interview at any time.

**Background information:**

- Have you ever been asked to complete a questionnaire about your health? Where? What kind of questions were they?

*Prompts: Did you provide that information? Was it in a clinical trial? Have you ever been asked to complete health questionnaire in routine clinical care? If so, in what clinical setting? Have any steps been taken to encourage you to complete* health questionnaire*? Have you been informed how this information can be used to manage your care?*

**Main questions:**

1. Would you be willing to complete health questionnaires to provide evidence on risks and benefits associated with treatment?

*Prompts: Do you think other patients would be?*

1. What would make you more likely to complete health questionnaires as part of your process of care?

*Prompt: Would you expect this information to be seen by your doctor and would this impact on your decision to complete?*

1. How often will you be willing to complete a health questionnaire? Would you be willing to use your own smartphone/computer to report PRO data?

*Prompt: Could you see challenges with this? Thinking about your friends and family would they be willing to do this – do your foresee any challenges for them or other broader members of society?*

1. How much time are you willing to spend on filling the questionnaire?

*Prompt: Are you willing to complete longer questionnaires if you feel questions are important to you?*

1. Would you like to receive reminders to complete questionnaire?
2. Do you have any concerns about providing PROs as part of your routine care?
3. Do you mind if pharmaceutical company would use your anonymised responses to test effectiveness of their products?
4. In what ways do you think medical teams can use the results of these questionnaires?

*Prompts: How well does it fit with how care is delivered now? What are likely issues or complications that may arise?*

1. What things would we need to consider in collecting this information?

*Prompt: whether it will inform their care, patient burden, relevance of questions to the patient*

1. Would you need support with providing PRO data? What kind of support?

*Prompt: What support might other patients need?*

1. Have you ever been involved in co-designing long term studies to ensure that drugs that have been approved for use are working as expected? Have you ever been involved in selecting a health questionnaire to be used in a study? What aspects should be considered when selecting it?
2. Do you think there is a need for patients to be given some training about the importance and how to complete these questionnaires? Are you aware of any training, resources or other forms of support to inform patients about PROs? How this could be improved?

*Prompt: If aware of the education campaigns are there more or less visible than campaigns targeting other problems?*

1. Do you have anything else to add?
