# Supplementary material for: Paving the way for patient centricity in real-world evidence (RWE): Qualitative interviews to identify considerations for wider implementation of patient-reported outcomes in RWE generation
Source: Heliyon. 2023 Sep 14;9(9):e20157. doi: 10.1016/j.heliyon.2023.e20157 (PMC10559915; doi:10.1016/j.heliyon.2023.e20157)
Supplement: Multimedia component 2 [file mmc2.docx]

Appendix 2. Other experts interview topic guide.

**Introduction:**

Introduce self as a UoB PhD student and that this interview is being undertaken as part of the research project funded by unrestricted educational research grant from GSK.

**Study recap (general purpose of the interview):**

Real-world evidence studies are used to assess the long-term effectiveness and safety of health interventions. Patient-reported outcomes could play an important role in this evidence base describing the impact of healthcare interventions on quality of life, daily activities and symptoms. Today, I would like to find out more about your perspective on current and future PRO use for RWE generation. The objective of this study is to better understand how different aspects related to PRO data collection, analysis and use should be approached to maximise the potential benefits of implementing PROs for RWE generation. I would also like to explore potential challenges to use of PROs in real-world evidence generation. PRO RWE data can be collected directly or remotely through various study designs, using questionnaires, mobile apps, telephone or being captured in patients’ health records.

**Consent**

Check that the respondents are still happy to take part and have signed the consent form. Participants will be reminded that all individual self-identifiers will be removed before transcripts are analysed and that they can stop the interview at any time.

**Background information:**

- Can I start by asking what your role is?
  - How long have you been in the post, what are your key responsibilities?
- Does your role involve collecting, using, or analysing PRO data?
  - If yes, what is your involvement? How do you or your organisation use PROs in RWE generation?

**Main questions:**

1. What do you think the value of using PROs in RWE generation is?

*Prompts: Can you compare it to other types of outcomes? Which areas would benefit the most by greater use of PROs for RWE generation?*

1. What are the most important barriers which hold back the full implementation of PRO data for RWE generation?

*Prompt: How these challenges might be addressed? Are infrastructure changes i.e. IT systems, staffing to support PRO usage, adaptions to existing workflow and care delivery systems needed? What about time and money needed? Can legal issues e.g. patient consent, data ownership be obstacles? What about willingness to collect/provide data by staff and patient, Missing baseline information, Missing data points?*

1. What would encourage/discourage the use of PROs in RWE studies?

*Prompt: What evidence supports or discourage the use of PROs for RWE generation?*

1. Can you describe how the PROs could be integrated into current RWE research/ regulatory process/reimbursement process?

*Prompts: How well does it fit with existing work processes and practices? What are likely issues or complications that may arise? What actions should be undertaken to minimise this burden?*

1. What aspects should be considered when selecting PRO instrument to be used for RW study?

*Prompts: Do you expect to see a preference for a particular type of measures e.g. symptom or generic QoL PROs? Who should be involved in this decision-making process?*

1. Do you feel that there is sufficient understanding and guidance on how PROs can be optimised in RWE?

*Prompt: In which areas is this lack of guidance most acute (if participant has identified a lack of guidance)? How should this lack of understanding/guidance be addressed?*

1. Thinking about your answers so far is there anything additional you would like to add from your organisational perspective?

*Prompts: Is your organisation planning to increase/promote the use of PROs in RWE generation?*

1. Do you have anything else to add?

**Other question if time allows**

Data collection

*Is primary or secondary use of PRO data for RWE generation more appropriate?* Are there any specific considerations that should be given to the mode of PRO data collection?

*Prompts:* *How these can be addressed?*

Should any special considerations be given about PRO data collection among underserved patient groups/ patients from diverse backgrounds?

Data analysis

Is a special approach for analysis of PRO data needed to enable RWE generation for regulatory, reimbursement or health policy?

*Prompts:* *Risk-adjustment for patient characteristics, pooling data across multiple health systems, missing data (single data point vs. multiple data points).*

Uptake of PROs

What kind of data quality requirements, policies, regulations, or guidelines can influence the decision to uptake PROs in RW studies?

*Prompt: At what level could it be introduced (local, state, national, international)?*

Who are the key influential stakeholders for the wider implementation of PROs for RWE generation?

*Prompt: What could be an efficient engagement strategy to get these people/organisations on board?*

Resources

Do you have sufficient resources to implement PROs for RWE generation? What costs need to be incurred to implement them?

*Prompts: Who should be covering costs associated with PRO data collection for the purpose of RW study?*

Education & training

Are you aware of any training, resources or other forms of support to help with PROs implementation? How this could be improved?

Are you encouraged to network with colleagues outside your setting?
