# Supplementary material for: Paving the way for patient centricity in real-world evidence (RWE): Qualitative interviews to identify considerations for wider implementation of patient-reported outcomes in RWE generation
Source: Heliyon. 2023 Sep 14;9(9):e20157. doi: 10.1016/j.heliyon.2023.e20157 (PMC10559915; doi:10.1016/j.heliyon.2023.e20157)
Supplement: Multimedia component 3 [file mmc3.docx]

Appendix 3. Summary of key findings – CFIR domains, belief statements and representative quotes.

| CFIR constructs | Themes/inner settings/individuals | Emergent issues | Illustrative quotation |
| --- | --- | --- | --- |
| 1. Innovation domain | | | |
| Innovation Evidence-Base  The degree to which the innovation has robust evidence supporting its effectiveness | Consultation duration | The use of PROs in routine practice does not prolong consultation time | 1. “Whether it prolongs the consultations and although I have data showing these things (PROs) don't prolong consultations, that's still the highest concern.” R2 |
|  | Clinicians’ reporting | Health Care Professionals’ (HCPs’) reporting misses some of the aspects which are important to the patients | 1. “If you just think about side effects, safety and tolerability from the patient perspective, there is so much literature that shows that physicians, doctors, and nurse practitioners are missing a lot of the picture (…) when they report.” R30 |
|  | Patient management | PROs help to deliver care which is appropriate to the patient’s needs | 1. “There were some signals around management. (..) The management improved. There were more appropriate referrals to other specialists.” R99 |
|  | Willingness to provide data | Patients are generally willing to complete PRO questionnaires | 1. “There’s (…) evidence to suggest that patients are willing to provide these data, if they’re going to be used.” R77 |
|  | Health outcomes | The use of PROs in routine care leads to better outcomes | 1. “I know studies that support its use in patient care, with (…) benefits for symptom control, for communication, survival...” R2 |
|  | Cost containment | The use of PROs in routine care generates savings | 1. “I've seen so many studies (…) where they're like: look how much money I saved.” R75 |
| Innovation Relative Advantage  The degree to which the innovation is better than other available innovations or current practice | General value statements | High potential of PROs collected in real-world across the entire healthcare decision-making | 1. “Depending on your perspective, there're tons of potentials.” R12 |
|  |  | PROs should not be overstated | 1. “It’s important to have a balanced concept about the usefulness of PROs. Because it’s important not to overstate their value, I think there are a lot of issues methodologically with them, that are still not completely well understood.” R100 |
|  |  | Lack of trust in non-clinical, non-randomised trials | 1. “I've actually stayed away from RWE type studies on the basis that I don't understand, and I don't have the confidence with data. (…) I always questioned use of that data because it is such a mess.” R74 |
|  | Applicability of PROs collected in real-world studies | Safety/tolerability monitoring | 1. “Certainly, one of the key areas is around tolerability. (…) In most clinical trials, (…) before marketing authorization we get good data on efficacy signals and (…) most clinical trials are powered for efficacy, (…) they're virtually never powered for the safety.” R85 |
|  |  | Can inform the individual care of a patient who provided PRO responses | 1. “You collect these PROs to then inform your clinic visit with the physician. (…) The physician is going to have it pop up on the screen before you walk into the room. (…) Oh, your pain is seven, OK, well, we’ve really got to take care of that today.” R30 |
|  |  | RWE informs care by showing real-world effectiveness of health interventions | 1. You could then feedback to clinicians and patients evidence as to (…) what actually happens in the real world, and that would have benefits in terms of clinical decision making, justifying new treatment approaches helping patients understand better what their future looks like.” R12 |
|  |  | Reimbursement decisions | 1. “There is value clearly in having patient-reported outcome measures to inform health technology assessments to better understand patients’ experiences and that data is routinely lacking in real world evidence sources” R80 |
|  |  | Descriptive RWD can inform phase III study set-up | 1. “Real world studies give you that flexibility to tap into some of those questions that you might otherwise overlook if you've jump straight into your phase III RCT.” R35 |
|  |  | Maximizing value of PRO data collection | 1. “If we’re going to be taking the time and investing the resources to collect PRO data we want to maximize its value and use it for as many different ways as we can to advance patient-centered care. (…) To demonstrate (them) to patients, you know they’re spending their time, and to demonstrate to institutions – they’re investing their resources, (that) is producing value.” R77 |
|  | Patient centricity | PROs improve communication with patients | 1. “This is really a communication intervention to try to improve symptom control.” R91 |
|  |  | PROs inform about patients' perception of their health | 1. “One added value is the PROs can give you information that you can’t get from other real-world data. Most of our real-world data is based on administrative databases, so we look at electronic medical records synthesised across patients, aggregated, pooled. So, we can see what procedures people get. We can see what doctors, they meet. We can look at how many hospitalizations they have. We can look at discharge diagnoses from those hospitalizations. We can see if they’ve been in the emergency room, etc. But we can’t get the patient's perspective on any of that.” R12 |
|  |  | A helpful self-diagnosis tool for the patients | 1. “It could possibly help them going forward as part of their treatment, (…) and even a self-diagnosis, I suppose.” R18 |
|  |  | PROs help to prioritise and bespoke care according to patients’ needs | 1. “Risk stratification is really important, because it means that the right patient gets the right treatment at the right time in the right location.” R16 |
|  |  | Strengthening the voice of underrepresented populations | 1. “It’s a voice that obviously has been completely underrepresented in the healthcare system, right? (…) PROs are a vehicle to get their information out there.” R75 |
|  | Information contained in PROs | PROs can provide a more complete picture on adverse events than from clinical report | 1. “If you look at the data from clinical trials and the adverse event data, you don't get a really complete picture of the adverse events. I mean if it's a grade 3 or 4 you do, of course, get that marking that this is a serious adverse event, but the nice thing with patient experience data, is that, it's being tracked along on a regular basis. You're able to see like: OK somebody reported severe diarrhoea which was then resolved by, because you have follow-up assessments, and so you see it getting resolved, whereas a clinician put in a note to the CRF that the grade three or four diarrhoea.” R30 |
|  |  | Some PROs inform about the impact of treatment on quality of life, which is a broader concept | 1. “We can have (…) kind of clinical binary, did it work or not, did it lower this lab value or not, that type of thing, but in terms of quality of life, is that actually helping the patient?” R83 |
|  |  | PROs has application in symptomatic diseases | 1. “There are some very serious diseases that have almost no symptoms (…) until you get closer to the end stage. Probably PROs are not all that useful there.” R100 |
|  |  | PROs are subjective but give a more complex picture than wearables | 1. “So that's the difference of wearables. People like them because they think that is objective. Count of what you're doing, but there's still a lot of subjectivity to it.” R30 |
|  |  | PROs help to understand some other types of RWD better | 1. “A lot of times, it is difficult for us to understand why a drug has been prescribed. There is no direct link to the indication. It is rare that we have it. (…). Maybe PRO could be helpful in these.” R26 |
|  |  | PROs demonstrate a more complex picture of the individual | 1. “Comparing to the other sources of RWD, so primary, secondary, registries, indeed a different type of outcome we can collect, quality of life gives a more comprehensive picture. Patient experience is what would add to the social data we already have access to. So yes, for us when we work on PRO data is having more outcomes and soft outcomes and having a broader picture.”R26 |
|  | RWE vs RCT | Broader populations and larger sample sizes | 1. “Generalise the findings to a broader population. We know that within the clinical trial setting, because of the many inclusion and exclusion criteria, the population is very defined, very small. What you kind of see with PROs or quality of life measures in these studies, it is quite limited to that specific populations.” R26 |
|  |  | Opportunity to collect data which were not captured in trials | 1. “One of the greatest utilities of real world data are to collect information that either can’t be collected in trials, or is deprioritised.” R75 |
|  |  | Informs about real-world effectiveness | 1. “You can do effectiveness studies of clinical drugs in the real world and find out whether they work as well in the real world, as they do in clinical trials. So, effectiveness versus efficacy.” R12 |
|  |  | Cheaper than trials | 1. “So, it's very expensive to collect PROs in clinical trials, that's a costly prospect, so, you know, it's one of the visions, that you could formally run the clinical trial say for five years and collect PROs on the trial and then, after that you could collect them in the real world and extend your effective follow-up” R12 |
|  |  | Provide information about various sub-populations | 1. “That would be better evaluation of a new drug and it will give you better data. Is it replicating the results from the trial, does it give completely different results, how does it work in other populations, 2. etc.? So, I think there is potential benefit in all of this.” R26 |
|  |  | RWE study can be conducted when a trial is not feasible | 1. “You can’t do clinical trials on everything, so we’ve learned a lot about how things work by doing natural experiments, you know, what’s done in one region (…) versus what’s done in the other region …” R12 |
|  |  | Can inform about subtle changes between treatment regimes | 1. “You have the potential to create a lot of outcome data with subtle differences in the way technologies are used. (…) It could be really informative to find out how different patterns of practice result in different outcomes from the patient's perspective.” R12 |
|  |  | Longer follow-up | 1. “The added value I currently envision is the longer follow up. (…) For example, (…) cancer is becoming more and more curable. It means that we end up with cancer survivors” R26 |
|  |  | Multiple sources of heterogeneity in RWD samples | 1. “That’s what makes it valuable in one sense because it’s so heterogeneous, and you’d hope that some sort of signal would arise up above all that noise.” R12 |
| Innovation Complexity  The degree to which the innovation is complicated, which may be reflected by its scope and/or the nature and number of connections and steps | RWD definition | Confusion about RWD definition | 1. “But what the hell is real world? I’m still struggling with that definition. What is that?” R74 2. “So yeah, I'm still not convinced, you can do real-world data studies with PROs to be real-real-world, because many patients will not participate... So, it's still better than a trial in terms of how generalizable it is, but it will never be you know exactly what (real-world is).” R2 |
|  |  | Only data collected as part of routine care | 1. “I think if someone is (…) use a wearable device, allow someone to monitor them and do that as part of a research program that to me is not real-world evidence. (…) That's research. (…) The real-world evidence is taking stuff that happens in the real world, not people that you convinced to wear a wearable device and monitor them and do research on that. That's a very selected subgroup of patients. That's not real world. Real world would be getting Google data or Facebook data to see what happens (…) in unselective people that have no idea that they're participating in a research.” R12 2. “If you’re selecting PROs specifically to collect real world data I think you’re missing the point that they ought to be used in clinical practice and then used to inform real world evidence (…). Else, all you’re doing is a broad based population research project, which is a different thing, right? (…) That’s not real world anymore. That’s to me broad based research project.” R12 3. “You still lose patients who don't want to do it - don't want to consent, and I think it is possibly tricky to actually collect that data without patient consent.” R2 |
|  |  | Everything outside of the clinical trial | 1. “It’s everything, but a clinical trial. (…) It’s a very broad definition. I think that is fine, in terms of the purposes of (…) how this can be useful for a multitude of stakeholders.” R8 |
|  |  | Primary vs secondary use of data | 1. “I think what's more important is to be clear on the source of the data, regardless of what you call it. So, regardless of whether it's real-world evidence or not real-world evidence, (…) (for example) you (can) have secondary data, so all of the PRO data (…) were collected for a different reason, for their primary reason and you're doing it for a secondary reason.”R77 |
|  | Ethics | Patients need to be sure of the purpose of PRO data collection | 1. “Having the data anonymised and non-traceable is important to patients, (…) because patients have to learn to build trust and there has to be transparency around the use of PROs. (…) They need to be clear that (…) PRO collection isn't going to be used for any purpose other than the purpose that they've agreed to initially.” R21 |
|  |  | Patient consent is not needed for universal data collection in routine practice | 1. “It's routine care, if it's everyone coming through the door, then that is part of your care and you do not need to sign a separate consent form” R91 |
|  |  | Patient consent needed to use their data for research | 1. “If you want to use it for research, you need to ask the ethics board for permission.” R99 2. “The portal asks for personal information, and then it asks the question of whether the patient will be interested in participating in research, and if so, these data will be collected. So, there's an ethical piece there that is associated with the ethical board in our institution.” R99 |
|  |  | No standards for obtaining patient consent in real world | 1. “How do you consent patients, what do you do to support patients in terms of making a decision (…) if they want to be part of the PRO study or using PRO data and how would that data be used (…) is absolutely essential and understanding the framework for delivering that content is equally really important.” R85 |
|  |  | Privacy issues apply to all types of RWD, patients should be informed of how data will be used | 1. “I don’t know that the PRO data are so different from any other data that would be used, but I do think we need to be clear and transparent with patients about how all of their data may be used or will be used.” R77 |
|  |  | Not using collected data is unethical | 1. “So, I think we have to be really thoughtful about (…) balance between collecting patient experience data and ensuring that we then use that data and we don't waste their time and efforts.” R30 |
|  | Data collection | Much messier data is collected in real world | 1. “(It) is a big data set, but it's often not quite clean; it's not like a data set you get in a clinical trial where statisticians go through and data managers.” R2 |
|  |  | No statistical methods will help when data of low quality | 1. “I just really want to emphasize the data fitness perspective. (…) It's not about the number of data points; it's about the quality of the data. (…) So, you could have the best analytics and understand what you want to study, but if the data aren't collected well, you're in trouble.” R75 |
|  |  | Extensive data management is needed | 1. “Patient-reported outcome, meaning they are self-reported, (…) so it's up to the willingness of a specific patient, whether or not to participate. (…) So, it requires a lot of data management.” R26 2. “Statisticians have the knowledge and how to impute data. How to improve the quality of data, how to account for potential biases or limitations of the data sets, how to set up the sample size, so your findings are reliable, right?” R26 |
|  |  | Multicountry data collection is challenging | 1. “And how do you collect real-world data across 12 countries in a standardised manner? It is impossible. So, it is very difficult. It's not an easy field to work in, I think.” R74 |
|  |  | ePROs increase the quality of collected data | 1. “Although with electronic PROs that's usually OK - the (data is of better)quality and less patients stop halfway and never continue.” R2 |
|  |  | Selection bias due to voluntary data provision | 1. “Your population may not be completely representative because there are certain characteristics of people who may participate in these things that are different from those who choose not to.” R83 |
|  | Data analysis | Missing data is the biggest issue | 1. “I think we would have to really think about how (to) approach missing data. Based on the timing of the data collection. So, I think that’s critical” R8 |
|  |  | Describe who is missing | 1. “So, we don’t have those tools (that are) used in the clinical trials. (…) It’s a huge problem. (…) If you could at least describe your entire population and describe from that who you’ve got data on, that’s a start.” R12 |
|  |  | Use appropriate statistical methods | 1. “Depending on what the data (type it) is, (…) ensuring that appropriate statistical techniques are used to analyse the data. So there would be situations where you could have (…) different reporting along those scales, but you could be able to use some type of statistical technique to adjust for that. (…) Always be ensuring that you are using the right tests for the right data.” R83 |
|  |  | Using data for secondary purposes requires more statistical work | 1. “If we want to use (data) for new research questions (…) then we have to be open to doing the behind the scene work that will make them up to the standards we would need.” R8 |
|  |  | Similar problems apply to other types of RWD | 1. “There's all the variability that applies, that I want to emphasise, applies no more to PRO data than it does to other kinds of real-world evidence, such as (…) varying time points, varying modalities of collection, varying circumstances where the data is being compared. (…) But (…) those things (…) apply to real-world evidence overall not specifically to PROs.” R77 |
|  |  | Level of required data robustness depends on future application | 1. “I think that there are different considerations when you look at the collection of PROMs for different purposes. So, If you look at it from the purpose of drug approval, (…) then I think the considerations are very different. And, in many cases, it’s like keeping the considerations as they are kept in a randomized clinical trial, where you want to get your data perfect” R99 2. “That depends on your application and how important that is; if you’re doing something more descriptive and understanding the population, it might be less important than if you’re trying to use this data to look at the kind of effects. And obviously, then you need to be doing some sort of risk adjustment process. And obviously, these PROs, when they’re not used as outcome data, can also be useful (…) to kind of better balance patient characteristics at the baseline if you do have this information.” R80 3. “However, we're not going to be analysing it in any kind of comparative way. We're going to be using it as a descriptive to look at trends over time for our patient population.” R35 |
|  |  | Statistical methods already exist and can be drawn upon from clinical trial settings, but there is need to set up standards for communicating results | 1. “There’s obviously a lot of literature on methods around missing data for PROs. I come across a lot of literature on that. It is often in the context of trials, but it does, I think, extends quite naturally.” R80 2. “Statisticians (…) can bring it easily from a methodological point of view; they can bring it to the RWD. It's just if you want to convey the added value of PROs you need to have a standard way of communicating results.” R26 |
|  | PRO methodology | Measurement situation impacts PRO results | 1. “There are a lot of issues methodologically with them, that are still not completely well understood, which is surprising. (…) The situation is the most important variable in measurement, right? So, when you think about the psychological situation (…) (you need to think) about the motivation of the subject, are they at all motivated by social desirability or are they motivated by the desire to malinger or, you know, not give a true answer exactly. So, that really needs to be considered, because PROs, they're not like biomarkers. (…) They can be measured with a lack of reliability.” R100 |
|  |  | It is not always feasible to use PRO due to the nature of the illness | 1. “I work in a lot with rare diseases and oftentimes, I cannot use a PRO. So, I'm actually more dependent (…) on a caregiver” R8 |
|  |  | The recall period can be problematic when collecting data in real world | 1. “There are some challenges, depending on recall periods and things like this, so if I ask you about your pain and ask you to recall the seven days, this is pretty different to asking you about your pain today.” R30 |
|  | RWE infancy | Significant barriers stopping PROs implementation | 1. “If it were easy, we would have done it a long time ago. And stopping dead and it's not been done, because of the tremendous importance of the barriers” R12 |
|  |  | Still challenging to assess the importance of PROs to RWE | 1. “I think we're still really early, and so I don't know that it has a value at this very moment which frustrates me” R30 |
|  |  | Although PROs are successfully used in RCTs, it a new concept in the RWE space | 1. “PROs these days have proven their point. You can see them as a secondary objective in clinical trials; they are knocking on the door of the RWD.” R26 |
|  | Barrier types | Operational and methodological barriers | 1. “I think probably most of the barriers are on the operational side. I mean there are methodological challenges which we can get onto, but I think it's more about embedding the kind of a consistent use of PROMs within data collections.” R80 |
|  |  | Secondary PRO data use is hindered due to limited data capture in routine care | 1. “Barriers to measuring PROs in practice (…) are upstream from the barriers to using them in the real world, a lot of them.” R12 2. “I think it's mainly data collection issues that might be the barriers.” R26 |
|  | PRO instruments | Multiple instruments used to measure similar concepts | 1. “When the PRO data are there, there’s the challenge that different measures are being used to assess the same thing and we have limited, although increasing ability to crosswalk scores among different measures.” R77 |
| Innovation Cost  The degree to which the innovation purchase and operating costs are affordable | PRO data collection as part of routine care | Expensive system-level implementation projects | 1. “I think the biggest challenge is going to be introducing real-world data collection into the routine practice. (…) Because there isn't the funding for that unless somebody's going to go behind it, like a pharmaceutical company.” R35 |
|  |  | Resource-intensive data collection | 1. “I think it’s very resource intensive, timewise and if I see how PROs questionnaires how they are collected – it’s very resource intensive.” R26 |
|  |  | No need for system-level implementations for some industry sponsored studies | 1. “It (…) depends on kind of study we're talking about. (…) So, if it's like data collected from the electronic health record, then it needs to be in place that they're routinely collecting that data. So, yeah, I would agree for something like that you would need to put in quite a detailed structural change for that department or whatever to be able to routinely (…) collect additional data that they otherwise weren't. So, for that, it would be very challenging. I think for other studies, whereby (…) you're almost setting it up like you do for a clinical trial, where you kind of get the site on board (…), you pay them for their time and collecting the data (…). But of course, you need a big machine there, which is normally the pharmaceutical industry.” R35 |
|  |  | It might not be possible to extrapolate the benefits of close patient monitoring to the system level | 1. “Those studies, while they have really great outcomes, show that they takes a lot of infrastructure and resources to pull that off. I don't know that anybody's like dive into how much the costs would be to scale it to the entire healthcare system.” R30 |
|  | Hospital-level cost of data capture | Investments need to align with benefits stemming from the implementation | 1. “There's an upfront cost for that, obviously - and then an ongoing cost and the upfront costs, this is larger because you do need the IT costs, you do need to pay a company to run it, then you potentially start saving afterwards, but it's all predictions, how much you'll save.” R2 |
| 1. Outer setting domain | | | |
| Critical Incidents  The degree to which large-scale and/or unanticipated events disrupt implementation and/or delivery of the innovation | COVID pandemic | COVID distracted healthcare systems from PROs | 1. Especially with COVID, we're trying to keep people alive instead of measuring their PROMs.” R12 |
|  |  | COVID constrained available hospital resources | 1. “It’s more resistance from the hospital IT departments that are busy with COVID and all of us doing the remote work etc.” R2 2. “There's a nursing staffing shortage in the US right now that has been made much worse by COVID, so we don't even have enough people in the clinic” R91 |
|  |  | COVID impacted patient lives, which is reflected in collected data | 1. “I haven't had GP’s appointment for three years because of COVID.” R3 2. “Patients have just not been completing their questionnaires because of illness or because they’re not doing their usual activities that they normally do, which then impacts your data because you’re looking at something like activity. You can’t even measure that, because they’re locked down.” R35 |
| Local Attitudes  The degree to which sociocultural values (e.g., shared responsibility in helping recipients) and beliefs (e.g., convictions about the worthiness of recipients) encourage the Outer Setting to support implementation and/or delivery of the innovation | How to use PROs for RWE generation | Not enough understanding of how to use PROs in the RWE generation | 1. “No, I do not think there's any understanding.” R85 2. “I think it's just that there's a lack of consensus within the field on how you would interpret PRO data in the real world setting” R85 3. “There's no standardised design, how do you standardise the studies, how you standardise the way it's collected.” R74 |
|  |  | Lack of agreement on how to collect data | 1. “I think there’s not necessarily a very visible framework in terms of how you would collect that data. It’s not clear who would fund the collection of that data, so unless it’s a mandated regulatory condition of a marketing authorisation which we do, we can ask companies to collect more data, unless it’s mandatory. Why would you do it, so there has to be an advantage for companies to be able to do it.” R85 |
|  |  | Lack of consensus about types of conclusions which can be supported with RWD | 1. “I don't think there's sufficient robustness in the understanding of exactly how we would use that data going forward for me. What decisions can we make based on that data. These data may be interesting, and it may be providing signals, but what type of follow-up do we need to do, based on that data? R85 2. “I would say we're probably in the infancy of utilising RWE and RWD (…) in terms of being able to support efficacy. Because we do have a regulatory standard, we have actual law in the US, of what has to be met to be able to make a claim about efficacy.” R8 3. “Depending on the context of use, there is variation how committees are willing to accept it and the confidence that they have in it. So, if you’re using it for comparison effects, there’s likely to be most scepticism or challenge to it, whereas if you’re using that as more characterisation of a patient group or perhaps like parameterising in an economic model or something, then it might be more accepted.” R80 |
|  |  | For some disease areas, PROs are less important than other types of outcomes | 1. “But for anti-cancer, you’re always going to have survival, progression-free survival, disease-free, all those things are going to be your primary and secondary endpoints. Patient-reported data will always be at the bottom of the endpoint hierarchy or an exploratory, supplementary information.” R30 |
|  |  | PROs are more important outside of comparative effectiveness studies | 1. “But if it’s not (…) effectiveness (…) and it’s more about understanding your patient population, then you have PROs as your primaries and your biological ones can come later.” R35 |
| Local Conditions  The degree to which economic, environmental, political, and/or technological conditions enable the Outer Setting to support implementation and/or delivery of the innovation | Collection of PROs in routine care | PROs are still not being routinely collected | 1. “At most venues, PROs are not routinely collected. So, you know, there is a lack of PRO data.” R77 |
|  |  | Lack of necessary infrastructure in place to collect PROs | 1. “But until you have a systematic infrastructure, where you try and collect these data in a systematic way to answer questions, it's just not going to happen opportunistically” R12 |
|  | Availability of PROs in RWD repositories | PRO data not available in RWD databases | 1. “These big data curation groups, they don’t have patient-reported outcome data.” R30 |
| Partnerships & Connections  The degree to which the Inner Setting is networked with external entities, including referral networks, academic affiliations, and professional organization networks | Collecting data from healthcare providers | Need cooperation form hospital health informatics teams | 1. “We've been begging for about a year, to get the data and then they made a small mistake in it, so we didn't get everything - another six months, and while we got their attention, it took them 10 minutes. But that is kind of how you work with big hospital departments that have other priorities. And home much you pay them, I suppose.” R2 |
|  |  | Data-sharing agreements need to be in place | 1. “Then you need to download the data, so that will get you through the data sharing agreements, all these contractual things.” R2 |
|  |  | Electronic data capture allows for interoperability between different databases | 1. “In that 15 years, every clinic has been gathering more and more data and less and less of it's on paper and more and more of it captured in computing systems. And more we're getting these computer systems to be able to talk together in new ways, and we are going to see an explosion of opportunity and PROs have got to be there.” R40 |
|  |  | The complex process, with the involvement of many stakeholders | 1. “Who downloads the data for you in that RWE (study)? Is it the IT department? So, I think it's a complex procedure and to look at each step of it.” R2 |
|  | Networking | Various stakeholders should network across the board | 1. “So I think like regulators, (…), HTAs and other payer societies, (…) professional societies, I think it would be so cool to really advanced space, you need to have some multistakeholder partnerships.(…). And then to also consult patients. So, I think you gotta be broad and kind of aim high, right?” R75 2. “I think that's why we need to work collectively, proactively, outside of drug development programs to really hone in on how can we best operationalise the collection of this type of data in the real-world setting. In a way, that could be used for those multiple stakeholders.” R8 |
| Policies & Laws  The degree to which legislation, regulations, professional group guidelines and recommendations, or accreditation standards support implementation and/or delivery of the innovation | Existing guidance | Existing guidance can be also applicable (to some extent) to RWE | 1. “I think there’s enough guidance out there. I mean there’s FDA guidance, there’s NICE guidance, so I think there’s enough guidance. It’s actually executing that guidance is the hard part.” R12 2. “I think there's sufficient, but I think as the field matures that it will advance, so I don't think we're the in a place where we have to say like we don't know enough to do it at all. I think we know enough to do it, I think, over time, will learn how to do it better.” R77 |
|  |  | Guidance for PROs in RWE generation is needed across the board | 1. “It comes down to partly around lack of consensus in the sort of requirements for that data collection, say in clinical trials you’ve obviously got (good clinical practice, we don’t have to the same level that sort of a framework for collecting data in the real world setting.” R85 2. “I mean, even a lot of the guidance which is out there is focused on trials, right? So, we definitely need a better intersection of like epidemiology and PROs.” R75 |
|  |  | Guidance helps the industry to generate meaningful evidence | 1. “When you put up standards and guidance, (…) you give them (industry) the confidence to do things like this, so that you can de-risk it where they can. Of course, ultimately, the evidence has to speak for itself, but let's help them at least try to generate meaningful evidence that is actually interpretable by a decision-maker, right?” R75 |
|  | Mandating PRO collection | Universal PRO data collection | 1. “A few years ago, they decided to implement patient-reported outcomes, I think, across all diseases, not just cancer, certainly in cancer across Denmark. All hospital sites became obligatory to start using it, and they are paid.” R2 |
|  |  | Regulators and payers can require data collection for a specific drug or disease | 1. “There are situations where we more directly require data collection, so it is as part of managed access. But in a regulatory context, you obviously have post-marketing surveillance studies and things like that.” R80 |
|  |  | A significant rate of missing data | 1. “Although it’s mandated to collect the PROs on every patient, we only get them in about 60% of patients.” R12 |
| Financing  The degree to which funding from external entities (e.g., grants, reimbursement) is available to implement and/or deliver the innovation | Financing PRO data collection | The way of financing highly depends on the goals and objectives of data collection | 1. “Who should pay for it? It depends on what it’s used for.” R80 2. “You need to make sure that the people who are benefiting from those efficiencies are the ones who are investing the money and collecting the data.” R77 3. “If people can’t be guaranteed they’re going to be reimbursed for this, it’s not going to happen.” R91 |
|  |  | Alternative cost of data collection | 1. “So, the government de facto is paying for it through the use of taxation. And so, if they spend money on collecting PROs they have less money to spend on something else, so you have to show the value.” R12 |
|  |  | Industry-sponsored prospective data collection | 1. “You're almost setting it up, like you do for a clinical trial, where you kind of get the site on board (and) you pay them for their time and collecting the data.” R35 |
| External Pressure  The degree to which external pressures drive implementation and/or delivery of the innovation. Note: Use this construct to capture themes related to External Pressures that are not included in the subconstructs below | Societal pressure | Increasing interest in utilising RWD for decision-making | 1. “I think the field is obviously moving quite quickly now.” R85 2. “There's a lot of effort in this space right now.” R30 3. “Obviously, there's been a big boom in the wanting to use real-world evidence to support question marks.” R8 |
|  | Market pressure | PROs are being used more widely in routine practice as healthcare providers duplicate workflows of similar facilities | 1. “When one department has used it for some years, then all departments with the same type of patients, they are often asking why we do not use it?” R25 |
|  |  | Regulatory bodies internalise similar regulations | 1. “If you say FDA also does it. (It is) like: Oh, then we also needed it.” R26 |
|  | Performance-measurement pressure | Some interest in using PROs for performance measurement, but a lack of benchmarks hinders use | 1. “So, they want to eventually, possibly move to a model where care is judged based on patient perceptions and not on quantity. (…) But we don't have any benchmarks yet. So, we don't know where people should be. So, what percentage of your chemotherapy people should be reporting nausea? Hopefully, it would be low, but we don't know what is low.” R91 |
| 1. Inner settings domain | | | |
| IT Infrastructure  The degree to which technological systems for tele-communication, electronic documentation, and data storage, management, reporting, and analysis support functional performance of the Inner Setting | Healthcare provider | Most healthcare providers have some Electronic Health Records (EHR) system in place and PROs could be integrated there | 1. “I think the IT infrastructure isn't that difficult (…) because (…) most hospitals have electronic records. You can collect patient-reported data using any software, any app and then all you need to do is link it to the electronic records integrated via a standard interface called API.” R2 |
|  |  | EHR system should be able to analyse data instantly and feed back results to the clinician | 1. “In medicine, you want to have somebody come in, fill out the form and have the results available to the physician for that visit, and that requires a certain infrastructure.” R100 |
|  |  | EHR systems have low usability for PRO data collection | 1. “It's a common problem that you buy a new EHR system and the sellers promise everything that you can do anything, including entering PRO, but this is definitely not the case or it is a very primitive way it can handle PRO.” R25 |
|  |  | Data collected as part of prospective studies usually are not fed back to EHRs | 1. “They'll use it for their research purpose, but it doesn't overflow into routine daily clinical care for everyone.” R91 |
|  |  | Multiple prospective studies collect data using different platform | 1. “So (one) organisation (…) develops something for their particular product, and then someone else does something for their particular product and then it is not particularly friendly for health professionals to use.” R41 |
| Work infrastructure  The degree to which organization of tasks and responsibilities within and between individuals and teams, and general staffing levels, support functional performance of the Inner Setting |  | Data collection needs to be integrated into the existing workflows | 1. There's a culture issue and a workflow issue. But both are addressable with effort.” R77 |
|  |  | Implementation is context specific | 1. “So, usually what we have to do is work with each clinic individually. So, even if there were two clinics in the same service line (…), but they were radically different on their workflow.” R91 |
|  |  | PROs compete with other tasks currently in the workflows | 1. “Additions to the process, really will compromise the other processes that are in place. So if, I have to talk to the patient about the PROMs results, I might have no time to talk about the X-rays results, because I have seven minutes visit with the patient.” R99 |
| Compatibility  The degree to which the innovation fits with workflows, systems, and processes |  | PROs are rarely routinely collected at the large scale with integration in the EHR | 1. “Nobody is (collecting PRO data routinely) in the UK in that setting. So basically, they thought it's happening and it's going in the electronic records, well it isn't. There may be one or two places, but it's not going into electronic record.” R2 |
| Funding  The degree to which funding is available to implement and deliver the innovation |  | Data collection is resource intensive | 1. “It needs infrastructure, money, people to be available to collect that data. So, a lot of work needs to be done on this and we are really at the early stages, I think.” R74 |
|  |  | Getting paid by a study sponsor is strictly regulated | 1. “In some countries, you're allowed to pay more, in others, there's quite a lot of restrictions on what you can pay a clinician.” R35 |
|  |  | It is a hospital investment to introduce PRO capture in EHR, so the return on it needs to be seen | 1. “It costs money to implement a system. So you have to have the folks at the top of the hospitals, who make these decisions, believe that this is worth the X thousands to implement a system.” R30 |
| IT infrastructure  The degree to which technological systems for tele-communication, electronic documentation, and data storage, management, reporting, and analysis support functional performance of the Inner Setting | Healthcare system | Implementation at the system level is a big infrastructural project, and it is impossible to find private sponsors for that | 1. “To integrate it with the health medical record to make it more useful clinically, that's a big big project and pharma companies not going to pay for that just to get the evidence on the one drug that they want to do the post marketing strategy.” R12 |
|  |  | Fragmented health informatics | 1. “One of the challenges is the fragmented nature of the health system in the sense that every different hospital has different systems in place.” R41 |
|  |  | A common system for data collection by multiple stakeholders would be useful | 1. “If you are a company and you want to collect data in the real-world setting, one of the questions you're going to ask is: well, how much is it going to cost? What systems can I use? Is there already a data capture system that I can plug into to be able to collect that data?” R85 |
| Incentive systems  The degree to which tangible and/or intangible incentives and rewards and/or disincentives and punishments support implementation and delivery of the innovation |  | Using PROs can be beneficial to the entire healthcare system | 1. “If you use clinic-based PRO tools to better manage and personalise the care for patients, you can keep many at home and not bring them into hospital, and you can promote better side effect management, disease symptom management, reducing the chance of E&A admissions, overnight hospital stays. You can improve compliance with treatment regimens, etc. That brings about huge efficiencies and benefits to the health system.” R73 |
|  |  | Health systems need to incentivise healthcare providers to collect data | 1. “That's a technology problem and an incentives problem.” R75 |
| Relative priority  The degree to which implementing and delivering the innovation is important compared to other initiatives | Industry | Collecting PROs is deprioritised through most of the drug development process until reimbursement starts to be discussed | 1. “I would shout with my flags: You need to do mixed methods, you got to be qualitative. They would never invest in it. Because they're too busy focusing on the primary and secondary endpoints. PROs come in as exploratory or lower secondaries. They are never powered sufficiently to get the data that you need to say anything of any value anyway.” R35 2. “You're trying to get it out to the prescribers, to the patients to the markets - so good price, then it's critical we have this data. And then suddenly there is this little gap from marketing authorisation to that. It's like: quick, quick, everybody run around and get this data, because now we need it. We're not going to even get the product over the line if we don't have essential data, especially in Europe.” R35 |
| Incentive systems  The degree to which tangible and/or intangible incentives and rewards and/or disincentives and punishments support implementation and delivery of the innovation |  | Incentives for drug development groups are closely linked with obtaining market authorisation, which is rarely dependent on PROs | 1. “Those groups (…) have got different endpoints they're trying to meet, and they are just trying to get that marketing authorisation.” R35 |
| Mission alignment  The degree to which implementing and delivering the innovation is in line with the overarching commitment, purpose, or goals in the Inner Setting |  | Companies need to see that RWD can help with their business objectives | 1. “I think drug companies need to see success stories. And when they will see drugs being approved, OK now I'm willing to put my toe in the water and take the risk.” R30 |
| Funding  The degree to which funding is available to implement and deliver the innovation |  | Efforts are needed to convince a company to sponsor the study | 1. “It's always a challenge to actually get a real-world study invested in and respected.(…) I'll propose a real world study and people will not understand the value of that.” R35 |
| Patient-centeredness  The degree to which there are shared values, beliefs, and norms around caring, supporting, and addressing the needs and welfare of patients | Payer | Payers seek to know more about patient experience | 1. “There's a lot more emphasis now on trying to get a broader sense of patient perspectives and experiences of the condition. So it's not just about that sort of cost per QALY type evidence, but also about patient experience to complement that evidence.” R80 |
| Compatibility  The degree to which the innovation fits with workflows, systems, and processes |  | Standards describing the value of RWE in reimbursement decision-making are needed to fully incorporate their use | 1. “For the use of PROs to kind of supplement (…) evidence, you might want a bit more clarity on exactly how that's going to be incorporated into the decision, what way it is going to be used. To kind of make people more confident that this data when it's collected is actually going to be used to improve care.” R80 |
| Relative priority  The degree to which implementing and delivering the innovation is important compared to other initiatives |  | Mandating PRO data collection as part of managed entry agreements can be the most immediate implementation of PROs | 1. “The area (…) where we are most actively using real-world evidence sort of routinely is in managed access. So that's probably the place to start.” R80 |
| IT infrastructure  The degree to which technological systems for tele-communication, electronic documentation, and data storage, management, reporting, and analysis support functional performance of the Inner Setting | Regulator | Regulators need access to RWD databases | 1. “But in the future, we'll probably have something that is more direct, so for DARWIN EU will have direct access to some of these data to the analysis” R26 |
| Work infrastructure  The degree to which organization of tasks and responsibilities within and between individuals and teams, and general staffing levels, support functional performance of the Inner Setting |  | Pre-authorisation RWE applications | 1. “When you have a drug submission in the pre-market area we consider real-world evidence as part of the (submission). It is seldom the pivotal piece of evidence, but it can be very complimentary to, for example, a clinical trial. (…) So, that's where it can help to inform that, but again the real-world evidence is just a piece of the puzzle.” R83 |
|  |  | Post-authorisation RWE applications | 1. “In the post-market environment, (…) if we're looking at information that we got through the vigilance database, we may supplement that also by looking through the literature or by initiating a drug safety and effectiveness network study with research teams, where we can collect that information. It provides that additional information so that we do get the patient perspective of their experience to take that into consideration in our decision making.” R83 |
| Patient-centeredness  The degree to which there are shared values, beliefs, and norms around caring, supporting, and addressing the needs and welfare of patients |  | Regulators seek to know more about patient experience | 1. “Regulators want to know what the patients really think and feel about a particular health condition. So, they're very important, in that way.” R100 |
| Learning-centeredness  The degree to which there are shared values, beliefs, and norms around psychological safety, continual improvement, and using data to inform practice |  | Regulatory bodies need to keep up with evolving field | 1. “There wasn't all that much new stuff (…)happening, but in the last 20 years it's been a lot of new developments. Things being borrowed from other fields: education, psychology etc.” R100 |
| Compatibility  The degree to which the innovation fits with workflows, systems, and processes |  | Extremally rare use of PROs for regulatory decisions | 1. “I think the main issue is just the lack of experience of using that data. It's something that we very, very rarely do. I mean, extremely rarely do. Even in clinical trials, often patient-reported outcomes are exploratory endpoints. And exploratory endpoints in most cases are not going to influence a regulatory decision.” R85 |
|  |  | Standards describing the value of RWE in regulatory decision-making are needed to incorporate their use fully | 1. “From a regulatory perspective, it's quite hard to know how to use that data or make recommendations how to use that data” R85 |
| Relative priority  The degree to which implementing and delivering the innovation is important compared to other initiatives |  | PROs collected in real-world can be used in the first instance for tolerability and safety monitoring | 1. “Now, in post-authorisation (set-up) we do have to rely on RWD, because the study is done, finite, it's close. So we need to see what's happening outside that clinical design environment and that's where RWD has a little bit better foot on the ground.” R26 |
|  |  | PROs collected in the real world can contribute to label expansion decisions | 1. “So I think about patient focus drug development and label expansion. I think we should be thinking about including more patient experience data that are generated from the real world.” R75 |
| Mission alignment  The degree to which implementing and delivering the innovation is in line with the overarching commitment, purpose, or goals in the Inner Setting |  | PROs can help to systematically answer regulatory questions | 1. “We couldn't support systematically our Committee in the decision-making, if we had no access to PROs. We could do it if we have access to primary care, secondary care (data), etc., and we can improve that answer through PROs. It wouldn't be the main source (though) for a systematical answer.” R36 |
| 1. Individuals domain | | | |
| Motivation  The degree to which the individual(s) is committed to fulfilling Role | Hospital managers | Hesitation to invest in PRO data collection at the hospital level, especially when future savings are uncertain | 1. “Business managers are not keen to pay, even we're talking about 20-30,000 GBP which, for a bit cancer hospital, isn't a lot actually. But they're not willing to do that. So, I think this is a barrier.” R2 |
| Capability  The degree to which the individual(s) has interpersonal competence, knowledge, and skills to fulfill Role | Industry employees | Lack of knowledge about the value of PROs | 1. “I had to educate every time I was in a new team. I had to educate people about the value of PROs. I think it's sort of a unique field that sort of more comes out of psychology or some other sort of ancillary field to medicine.” R100 |
| Motivation  The degree to which the individual(s) is committed to fulfilling Role |  | Involve PRO champions in drug development teams | 1. “You can put your PRO specialist into the trial development and they'll keep banging on the door to get this done. Because it's important for their objectives. They probably won't get a label claim, which will be probably how they will be judged, because that's always ends up being too far down on the list of clinical trial endpoints. But the rest of the team might not be at all focused (on it), they will be more core scientists who are looking at like safety endpoints and biometric stuff they really need to show it is efficacious.” R35 |
| Capability  The degree to which the individual(s) has interpersonal competence, knowledge, and skills to fulfill Role | Statisticians | Statistician involvement less present in the real-world setting | 1. “The clinical trial by default comes with statisticians. Statisticians that have knowledge how to impute data. How to improve the quality of data, how to account for potential biases or limitations of the data sets, how to set up the sample size so your findings are reliable.” R26 |
| Opportunity  The degree to which the individual(s) has availability, scope, and power to fulfill Role |  | Statisticians are often focused on other types of outcomes | 1. “Often, especially if the drug doesn't succeed or on the other hand, if it is succeeding and there's a rush towards launch, there aren't the resources to analyse the PRO data. I think that's probably changed since I left the industry, but it's may still be there.” R100 |
| Opportunity  The degree to which the individual(s) has availability, scope, and power to fulfill Role | PRO researchers | Not enough PRO experts | 1. “I definitely don't think we have enough people. I feel that our PRO world is very niche. There's only a small group of us.” R8 |
| Capability  The degree to which the individual(s) has interpersonal competence, knowledge, and skills to fulfill Role |  | Lack of epidemiological knowledge, which is essential to interpret real-world studies correctly | 1. “Not all PRO researchers have a background in epidemiology. That's needed to understand bias and all these things. I mean, epidemiology is really all about real-world studies.” R100 |
| Opportunity  The degree to which the individual(s) has availability, scope, and power to fulfill Role | IT specialists | IT specialists too busy with other projects, so challenging to get their attention on the PROs collection | 1. “They're just overwhelmed. I could offer them $50,000 but it wouldn't get me very much and it's not going to pay for long-term solutions.” R12 |
| Need  The degree to which the individual(s) is committed to fulfilling Role | Administrative staff | The needs of admin staff to help with better questionnaire administration should be identified | 1. “But no one ever really bothered to check with the front desk people about what would be helpful to them.” R91 |
| Capability  The degree to which the individual(s) has interpersonal competence, knowledge, and skills to fulfill Role |  | The quality of collected data depends on them | 1. “It entirely depends on the staff at the site completing the data of patients actually doing PROs.” R35 |
| Opportunity  The degree to which the individual(s) has availability, scope, and power to fulfill Role | Nurses | They can play an important role in data collection, but training needs to be offered | 1. “I think nurse practitioners are probably going to allies. (…) Because they're just have a higher touch rate with patients than the physicians and probably way you can get some culture change. But yeah, I do think you do need to have education…” R30 |
|  |  | Some resistance can be seen to the extra work associated with data collection | 1. “Patients were happy to fill it out. It was just that the nurses were the one that were objecting to it – it takes too much time, patients are so burdened. That's not true, patients actually didn't have a problem with it. It was them who had the problem.” R100 |
| Capability  The degree to which the individual(s) has interpersonal competence, knowledge, and skills to fulfill Role | Physicians | Variable level of PRO knowledge between different areas of medicine | 1. “I don't know that physicians are trained in use of PROs now. I will also say that I've worked in some areas, for example, urology, where PROs are really the primary endpoint, right? So, they really understand PROs.” R100 |
|  |  | Most physicians have problems with the interpretation of complex PRO concepts | 1. “And what do these scales and scores really mean? And can you generalise them? There's a very nuanced background that you need.” R74 |
|  |  | IT tools can flag to physicians reports, which need their attention | 1. “The massive benefit of ePROs is (…, that) we can use technology to say: OK, (…) which of the responses have changed and let's flag that one, which of the responses is significantly worse - let's flag the one as well.” R41 |
|  |  | Should be offered training on data collection | 1. “I think they might need some training as well, right? In terms of how to record, make sure that they are collecting these outcomes in an appropriate manner.” R83 |
| Opportunity  The degree to which the individual(s) has availability, scope, and power to fulfill Role |  | Lack of physician buy-in impacts the completeness of data | 1. “That's just a mindset of some clinicians that PROs don't add value to their practice. (…) So that increases the missing data, because, eventually, their patients stop filling them in, because they realise that the clinician isn't interested in looking at them. So they stopped filling them in. This is well described phenomenon. That's a problem. So, the clinician’s willingness to collect data in practice gets in the way of using those data in real world evidence…” R12 |
|  |  | Too many PRO alerts put-off clinicians from using it. Smarter algorithms are needed | 1. “I also think we need to get much smarter about alerts in electronic health record systems. So, i'm working with a new clinic (…) they're pushing back, because they don't like how many alerts they get. So, they get alerts for fatigue, which is not helpful.” R91 |
| Motivation  The degree to which the individual(s) is committed to fulfilling Role |  | Co-designing the study increase motivation | 1. “Inviting them to be co-developers could help from the beginning. If it's a top-down kind of thing, I think they're going to be more resistant.” R91 |
|  |  | Concerns about prolonging consultation | 1. “And then minimal involvement, I think, for clinicians is key. At least for oncologist.,They will appreciate it, when they have access to the data. But that's probably not their highest priority, whether it prolongs the consultations and although I have data showing these things don't prolong consultations, that's still the highest concern about it.” R2 |
|  |  | Perception of PRO value determines willingness to collect PROs | 1. “For the healthcare professionals, it depends on what they see as the value of collecting that data and if it's for subsequent use by someone like NICE in order to make decisions, it might seem to have slightly less relevant than using it for direct sort of clinical care, but obviously keeping them informed and getting buy-in about the purposes of this collection and why it's important (is key).” R80 |
|  |  | To improve care at the individual level | 1. “They're collecting it as part of clinical practice, so they're trying to derive benefits in their practice, either on the day of the encounter or monitoring people between visits. But their mindset is, how can I improve the care of this patient, not how can I do a research project.” R12 |
|  |  | To identify patients who need help | 1. “Many of them think it will help reduce patients coming to hospitals, long waiting lists, etc. While still help identify patients who need help and others that can self-manage.” R2 |
|  |  | To find out how treatment affects patients | 1. “I think they would want to, because I think, they know it would help them know how their patients are doing.” R8 |
|  |  | Champions help to convince other HCPs about PROs’ value | 1. “You need somebody in the clinic who believes in this, like a leader who believes in this. (…) You need somebody in there, showing that this has value for something (…) and then, others will come along. (…) You really have to be (…) passionate about it.” R30 |
| Need  The degree to which The individual(s) is committed to fulfilling Role | Patients | PROs help to inform about individual’s needs in broader perspective | 1. “We need to think about different ways of measuring some of those things to include with PROs, so things like climate change that affects her health, environment, the psychosocial stuff that affects us on a day-to-day basis.” R21 |
|  |  | PROs put patients at the center | 1. “Well, if we're putting patients at the center, we need to understand and learn about the patient and what their needs are.” R21 |
|  |  | PROs inform about the subjective impact of the disease on the patient | 1. “Somebody feels more or less pain, everyone's perception of things is different, and that's OK as well. But (…) maybe others could manage in that situation. But that person can't. (…) That just justifies that they need a bit of extra help or whatever, and that's the whole point of patient report rather that the ticking boxes.” R16 |
| Capability  The degree to which the individual(s) has interpersonal competence, knowledge, and skills to fulfill role |  | Need to find a balance between burden to the patient and collecting information of interest | 1. “Again, my concern all the time is to try and minimise the amount of time patients have to commit to complete questionnaires while still getting the data you need. And I think that's forever a challenge.” R40 |
|  |  | Irrelevant questions asked repeatedly pose an enormous burden to patients | 1. “Our experience is that the number of questionnaires is not actually normally a burden, burden is irrelevance of questions when they don't fit the circumstances in which they're being used. Often, they're too generic and therefore patients are being asked questions that just don't fit their situation.” R41 2. “One of the biggest things we hear from the burden perspective are people filling in the same questions where they're irrelevant, and when nothing has changed.”R41 |
|  |  | Computer adaptive testing (CAT) can reduce patient burden by asking more relevant questions | 1. “One of the things I'd like to see adopted more is a computerised adaptive testing.” R100 |
|  |  | Using PROs for some people can be easier than talking about their health | 1. “Sometimes people will prefer to write it down rather than speak about it, because some people find it difficult to speak about their illnesses.” R3 |
|  |  | The level of IT literacy needs to be considered when planning electronic data capture, but most of the responders should be able to use technology | 1. “Over recent years, the percentage who would not use technology at all has significantly decreased, and that's across all age groups. And obviously, that's cultural and country-dependent. So, I think the first point to say is not to overestimate the group that you assume would ask for a hard copy. Often, it's actually not the case. And even in the oldest age groups.” R41 |
| Opportunity  The degree to which the individual(s) has availability, scope, and power to fulfil role |  | People should be given an appropriate amount of time to respond. Remote data collection help with that. | 1. “There is more flexibility with (electronic data capture).” R16   “Once you've got your own time to read it and think about it, and do it, rather than (…) trying to do at the doctors so they're waiting for you to do it. And I know, sometimes the first answer - quick ticks are best, but sometimes you think: Well, hang on a minute! What? How does that affect me? And if there's more thinking, that needs to go to it... So, I think (…) if you've got that on an app that you can do it at home (it helps).” R16 |
|  |  | HCP buy-in impacts patient’s commitment to data provision | 1. “I think doctors can do a lot of encouraging and unconscious discouraging at times, so what you want is, you want the clinicians who are going to be giving the questionnaires to be very committed to the idea that they're gathering valuable data.” R40 |
| Motivation  The degree to which the individual(s) is committed to fulfilling role |  | Patients are generally happy to complete PROs | 1. “Patients are very keen to be listened to if they have something like I am diagnosed with something, even a little thing or I have a fever or something. ”R26 2. “The cancer patients are generally willing to contribute and participate. They're grateful that we look after them.” R2 3. “They want someone to hear their experience.” R8 |
|  |  | Altruistic motivation to help with research and improve the care of others | 1. “But to me and for many sharing data if it helps somebody, or if it helps, enables research, they would do it.” R3 |
|  |  | Being informed about the purpose of data collection and study progress increases willingness to participate | 1. “If they got a proper understanding of why the information is being collected and how it could possibly help them going forward as part of their treatment.” R18 |
|  |  | Patients need to be reassured that their data are safe | 1. “There’s like the privacy question: where's my data going and all that sort of things.” R16 |
|  |  | Lack of trust, especially among underserved populations | 1. “In the US there are groups of folks that have trust issues with healthcare, so they will be more hesitant and we know that, we are very much aware of that and we try to work with that.” R8 |
|  |  | Co-designing the study increases willingness to participate | 1. “We try to design studies that are really patient relevant and meaningful by using patients in our design phases. And so, we've changed the modality in which we collect that data also to make sure that it is fit for purpose and relevant to the groups. So, I think it's designed in that way you're reducing your challenges in that domain.” R35 |
|  |  | Results should be fed back to the patients | 1. “And I think what I really want to see more of, is being involved from the beginning to see it through right until the end and get the results. Often that doesn't happen. You fill something out. That's it. No feedback…” R3 |
|  |  | The fact that PRO will be used to inform their care is increasing motivation to participate | 1. “I am interested in providing this personal information because I understand that my care is going to be better and my health probably will improve or my survival would be lengthened.” R99 |
| 1. Implementation process domain (The activities and strategies used to implement the innovation. Distinguish the implementation process used to implement the innovation (activities that end after implementation is complete) from the innovation (the “thing” that continues when implementation is complete) | | | |
|  | Implementation priority setting | HCPs should point out populations and settings where PRO data collection can bring the most significant benefits. | 1. “We think it's the best (if it comes) from the clinician that, they see a place where it is good to use PRO. It's better than when it comes from top to bottom,” R25 |
|  | Pilot studies | Conduct pilot studies to identify the most important barriers | 1. “I think there are groups who are doing these sort of pilot studies to try and show, and even if you can't get a fully useful thing (…) you can sort of show where the pain points are.” R30 |
|  | Sustaining benefits in the long-run | Create a sustainability plan to retain the long-term effects of PRO implementation | 1. “Present a project as an initiative that is there to stay with a clear sustainability plan (…) Almost like a plan, these are the inputs, this is what I'm going to do. I need this type of support from you, the cost of that support, so if it can have an estimate right. And then, these will be the outcomes and then the most proximal outcomes will be X. And then long-term outcomes.” R99 |
|  | Setting up standards for PRO data collection | Data collection in routine care needs to be carefully planned and agreed upon | 1. “It starts with a vision and a program and then, you can ask: do we have the right IT to support this program? Right now there's no vision, there's no program. There's just a desire to collect PROs in clinic and then maybe use them in the real world, but that's too soften and mushy. You don't get quality data with an opportunistic approach.” R12 |
|  | Instrument design | Platforms for ePRO data collection need to be user-friendly and work across different types of devices and operating systems | 1. “Make sure whatever software we use, it works across different kinds of devices” R41 2. “So they need to be simple, precise, really.” R21 |
|  |  | PRO questionnaires usability should account for different disabilities patients might have | 1. “Yellow and green highlight is very good for visually impaired people.” R3 2. “And link to an audio that can speak it over like: “Question one”, and you can hear somebody literally hear what question one is.” R3 |
|  |  | Automatic reminders should be used to increase completion rates | 1. “And one important thing to make it easier - send reminders. Otherwise, people forget. So, if you want that data regularly, you have to remind them.” R2 |
|  | Instrument selection | Validated PRO instruments should be used if exists. If not, additional work needs to be done to check their measurement properties | 1. “Whether it's even valid in the population, you're always going to have that. You might have done some legacy work, like we've done work to show that, even though our PROs are not disease specific, we've done quite a lot of work on the psychometrics to show that they are actually robust and reliable and valid within our population.” R35 |
|  |  | PRO instruments need to be able to address the research question | 1. “I think it very much depends on the research question. Always your research question should address what problem or what questions you want to answer and then you would map you methodology and your endpoints and instruments to that research questions.” R85 |
|  |  | PRO instruments need to ask questions which are relevant to the patients | 1. “Questionnaires need to be brief. (…) You don't want to go: not applicable, not applicable, not applicable, not applicable, through a whole stream of questions. That 's discouraging.” R40 |
|  | Engaging | Engage stakeholders across the board Consider their involvement in the co-design of the study and target them with various educational endeavours | 1. “I think it's going to take educating, and I say this because we were confronted with this right now ourselves. It is educating about what is your purpose, how do you make a thoughtful approach.” R8 |
|  |  | Buy-in from health institutions is key for successful implementation | 1. “The main barriers are, we know, the institutions - the health institutions that are quite rigid and the time. So, because the institutions are very rigid it takes time to break that, you know, to soften up the rigidity and allow for these things to be integrated” R99 |
|  | Type of RWE studies with highest potential to use PRO data successfully | Start with prospective data collection and focused research question | 1. “I think, the secondary uses is harder, maybe just have too much noise, too many unknowns.” R30 2. “Well, you don't just go out and gather vast quantities of data for the sake of it. You know that's a pointless exercise. You achieve very little. When (…) you prospectively gathering data, you know the purpose you are aiming to put that data. So, you don't want big, loose general questionnaires. You want focused questionnaires.” R40 |
|  |  | Start with secondary use of data collected in routine care | 1. “I just think that to me the implementation and the integration of patient reported outcomes and patient reported experience outcomes into the healthcare systems to inform quality improvement, research, management is a given and it's happening. And then the use of this information, once you have it, through the health care system why the pharma companies need to go back and collect it?” R99 |
|  |  | Start with post-authorisation safety monitoring | 1. “I think it's important to say that the setting of where RWE can have an impact, currently is mostly in the post-authorisation setting where drug has already got a marketing authorization.” R85 |
|  | Setting up standards for the use of RWE | Decision-makers need to show that RWD are used and expected | 1. “I think it's like a concerted effort by decision makers, by FDA, by an HTA, by payers to say: we are using this information and we actually expect it.” R75 |
|  |  | The value of RWD need to be demonstrated by practice-changing studies | 1. “I think people need to see the added value of it. And it's not been shown yet. So, any of those things that can all be done at the same time. Consensus - right to standards and then you know practice changing studies that can be shown to everybody.” R74 |
|  |  | Decision-makers need to know how RWE can influence their decisions. Case studies would be helpful | 1. “Then it seems a bit we have it funny for us to encourage data collection, that we didn't understand how it might influence our decision making, so that'd be a reluctance from industry to spend the money. I think it's a bit chicken and egg and I think it comes back down to, you know, which I keep going on about i'm afraid, but it is about having some good examples about where the collection of PRO in real world evidence has made a difference. Because until you can demonstrate that it's added value or it actually has a use, then I don't think the field is going to move on, because there's no incentive or motivation for it to move on.” R85 |

Legend: R followed by a number represents the ID number of an individual participant
